# Effects of specific inspiratory muscle training combined with whole-body endurance training program on balance in COPD patients: Randomized controlled trial

**DOI:** 10.1371/journal.pone.0257595

**Published:** 2021-09-23

**Authors:** Bilel Tounsi, Amal Acheche, Thierry Lelard, Zouhair Tabka, Yassine Trabelsi, Said Ahmaidi

**Affiliations:** 1 Laboratory of Exercise Physiology and Rehabilitation (APERE, UR-EA 3300), Sport Sciences Department, Picardie Jules Verne University, Amiens, France; 2 Research Laboratory of Exercise Physiology and Pathophysiology: From Integral to Molecular Biology, Medicine and Health (LR19ES09), Faculty of Medicine of Sousse, University of Sousse, Sousse, Tunisia; Prince Sattam Bin Abdulaziz University, College of Applied Medical Sciences, SAUDI ARABIA

## Abstract

**Purpose:**

This study aims to assess the effect of inspiratory muscle training (IMT) combined with endurance training (ET) on balance in patients with chronic obstructive pulmonary disease (COPD).

**Methods:**

We studied 32 male patients (62 ± 6 years) with moderate to very severe COPD. They were randomly assigned to an experimental group (IMT+ET) n = 16 or a control group (ET) n = 16 with similar characteristics. The evaluations were carried out at inclusion and after eight weeks of the training period. Functional balance was assessed by the Berg Balance Scale (BBS), the Timed-up and Go (TUG), the Single Leg Stance test (SLS), and the Activities-specific Balance Confidence (ABC) scale. The strength of the inspiratory muscles (PI_max_) was assessed by maximal inspiratory mouth pressure. Functional exercise performance was assessed by the 6 minutes walking test (6MWT). IMT program consists in performing two daily sets of 30 inspirations with 50% of PI_max_ increased by 10% every two weeks. ET program consists in performing 30 min treadmill exercise at 60% to 80% of the average speed achieved during the 6MWT three days per week.

**Results:**

After the training period, the experimental group demonstrated greater improvements in BBS (IMT+ET vs. ET; p = 0.019), and in ABC (IMT+ET vs. ET; p = 0.014). However, no significant differences between groups were observed for TUG, SLS, and 6MWT. There was a significant difference between groups in PI_max_ (IMT+ET vs. ET; p = 0.030). Significant moderate correlations were obtained between ΔPI_max_ and ΔBBS for both groups (IMT+ET: r = 0.624, p = 0.010; ET r = 0.550, p = 0.027) as well as for ΔABC but only in the experimental group (IMT+ET: r = 0.550, p = 0.027).

**Conclusion:**

Compared to ET alone, the results suggest that IMT combined with ET enhances inspiratory muscle function and functional balance according to BBS and ABC in patients with COPD. We suggest that inspiratory muscle training might be introduced as additional training to pulmonary rehabilitation programs aimed at improving balance in COPD patients.

**Trial registration:**

**The trial registry name:** Clinical Trials; **Registration number:** NCT04084405; **URL:** https://clinicaltrials.gov/ct2/show/NCT04084405.

## Introduction

Chronic obstructive pulmonary disease (COPD) is characterized by persistent airflow limitation and respiratory symptoms, which are due to airflow or alveolar abnormalities [1]. In addition to pulmonary dysfunction, COPD patients develop other health disorders such as peripheral muscle dysfunction, cardiovascular comorbidities, systemic inflammation, and depression [2, 3]. Combined with aging, COPD comorbidities, especially peripheral muscle atrophy [4] and peripheral muscle weakness comparing with healthy peers [5], had a significant impact on falls in patients with COPD by increasing both the number of falls and the fear of falling [6]. These consequences lead to reduced activity, agility, strength, balance, and often bring about a loss of functional independence during normal daily activities [7].

The ability to maintain stability and balance are complex skills which are essential for independent mobility and avoiding falls [8]. Emerging data show that patients with COPD have significant deficits in postural and balance control that may be associated with a high risk of falling in these patients especially when compared with healthy age-matched controls [9–14]. In addition, it was demonstrated that trunk instability might negatively influence postural control in COPD patients [15].

In healthy subjects, respiratory kinematics have a disturbing effect on posture, but its impact is worse when respiration involves the rib cage rather than the abdomen [16]. Therefore, the diaphragm and the intercostal muscles are not only necessary in breathing function, but they also have a prominent role in performing postural tasks [17–20]. The stabilizing action of the diaphragm acts both directly, by continuous co-contraction contributing to postural stabilization during tasks that repetitively challenge trunk posture, and indirectly, by increasing intra-abdominal pressure to support the spine [18, 21]. In addition, postural drive and inspiratory drive depolarize the same motoneurons, and the postural contraction of the intercostal muscle alters their output during inspiration in a direction-dependent manner [17].

The weakness of the diaphragm and intercostal muscles is well-known in COPD patients [22]. Impaired postural stability has also been shown related to inspiratory muscle weakness in COPD patients [15]. These authors showed more deficits in postural stability in COPD patients with inspiratory muscle weakness compared to healthy control subjects [15]. Indeed, COPD patients had worse static and functional balance than healthy controls [23]. Balance disorders were more frequent in COPD patients than in matched healthy subjects while performing dynamic activities, and the incidence of falls in COPD was higher [14]. Severe COPD is associated with an impaired ability to recover balance and higher trunk muscle activity during postural challenges [24]. This increased trunk muscle activity may limit the contribution of trunk movements to balance recovery and may contribute to an increased risk of falls [24]. Indeed, it was shown that diaphragm movement during tidal breathing in standing position was significantly higher and faster in COPD patients compared to healthy subjects [25].

Therefore, inspiratory muscle training was found to increases the endurance and strength of the inspiratory muscles and to improve exercise capacity in COPD patients [26]. Recently, two months of home-based inspiratory muscle training showed to improve inspiratory muscle function and functional balance in community-dwelling older adults [27]. Strategies such as pulmonary rehabilitation, based on endurance training and strengthening training, were assessed to target balance deficiency in COPD patients [28]. However, significant benefices were observed when endurance and strengthening training were combined with specific balance training including proprioceptive exercise [29–31].

To the best of our knowledge, this study is the first attempt to investigate the additional benefits of IMT on balance in COPD patients. Based on these considerations, we hypothesis that inspiratory muscle training (IMT) combined with endurance training (ET) allows a significant improvement on functional balance in comparison with ET alone. Therefore, this study aims to evaluate the additional effect of strength gain of inspiratory muscle induce by IMT combined with ET on balance in COPD patients.

## Materials and methods

### Study design and subjects

This Randomized controlled trial used a parallel-group design. Moderate to very severe COPD patients attended our pulmonary rehabilitation center. They were randomized to a standardized endurance training (ET) program (active control group n = 18) or a standardized ET program with an addition of inspiratory muscles training (IMT+ET) (experimental group n = 17) using a computer-generated random number schedule with block sizes of two to six. A researcher who was not involved in recruitment or assessment performed randomization sequences. Assignment to intervention groups was concealed from all investigators to carry out the interventions or patients’ involvement. The allocation was done using consecutively numbered opaque sealed envelopes, opened after completion of baseline assessment with the presence of the patients.

Only patients with forced expiratory volume in 1 s (FEV1) <80% predicted and FEV1 / forced vital capacity (FVC) <70% would be eligible to participate in the study. They were between 45–75 years of age, and clinically stable. Exclusion criteria consist of (1) cardiovascular problem (2) diagnosed psychiatric or cognitive disorders, (3) progressive neuromuscular diseases, (4) severe orthopedic problems with a significant impact on daily activities, and (5) prior inclusion in a rehabilitation program (<1 year).

The study was approved by the research ethics committee of Farhat Hached Hospital of Sousse, Tunisia. The trial was registered in Clinical Trial under the number (NCT04084405). This study complied with the declaration of Helsinki. All participants gave written consent before they participated in this study.

### Intervention

#### Endurance training program

The endurance exercise training consisted in an 8-week program with three sessions a week for both groups (Experimental = IMT+ET and Control = ET) (Table 1). This part of the training program consists in aerobic supervised exercise training. The training consisted in 30 min of supervised treadmill exercise per session. Each session ended with upper and lower limb stretching. Each participant received an individualized program based on 60% to 80% of the average speed achieved during the six-minute walk test [28].

**Table 1:** Design of training program.

Groups	1^st^ 2-Week	2^nd^ 2-Week	3^rd^ 2-Week	4^th^ 2-week
**Control group (ET)**	**ET**	**ET**	**ET**	**ET**
	**Repetitions:** 3 d/wk.	**Repetitions:** 3 d/wk.	**Repetitions:** 3 d/wk.	**Repetitions:** 3 d/wk.
	**Duration:** 30 min of treadmill	**Duration:** 30 min of treadmill	**Duration:** 30 min of treadmill	**Duration:** 30 min of treadmill
	**Intensity:** 60–80% of 6MWT average speed	**Intensity:** 60–80% of 6MWT average speed	**Intensity:** 60–80% of 6MWT average speed	**Intensity:** 60–80% of 6MWT average speed
**Experimental group (IMT+ET)**	**+ IMT**	**+ IMT**	**+ IMT**	**+ IMT**
	**Repetitions:** 7 d/wk	**Repetitions:** 7 d/wk	**Repetitions:** 7 d/wk	**Repetitions:** 7 d/wk
	**Sets:** 2 × 30 inspirations	**Sets:** 2 × 30 inspirations	**Sets:** 2 × 30 inspirations	**Sets:** 2 × 30 inspirations
	**Rest between sets:** 5–10 min	**Rest between sets:** 5–10 min	**Rest between sets:** 5–10 min	**Rest between sets:** 5–10 min
	Intensity: 50% of PI_max_	Intensity: 60% of PI_max_	Intensity: 70% of PI_max_	Intensity: 80% of PI_max_

ET = Endurance training. IMT = Inspiratory muscle training. D = days. Wk = week. PImax = maximal inspiratory pressure. 6MWT = Six-minute walk test.

#### Inspiratory muscle training program

The inspiratory muscle training program was performed and monitored following previously recently published protocol [32]. The training was performed once a day, i.e., 7d/wk for eight weeks, using a handle device (PowerBreathe® Medic, IMT Technologies Ltd, Birmingham, UK). The training consists in making two sets of 30 breaths (4–5 min/set) with 5–10 min of rest between each set (Table 1). The Respiratory resistive load was set at 50% of the initial PI_max_ and then increased by 10% of the initial PI_max_ every two weeks of training, i.e., 50%, 60%, 70%, and 80% of PI_max_ [33]. Part of the IMT was performed and well instructed in the pulmonary rehabilitation center (3d/wk) for 8 weeks, and the other part was home-based training [32].

Patients are instructed to emphasize the use of their diaphragm and to ensure that their abdomen “sticks out” during each inspiratory manoeuver [34]. They were familiar with diaphragmatic breathing by sitting upright on a chair and placing one hand on the abdomen and the other hand on the ribs along the anterior axillary line. The subjects then breathed and keep their hand on the stationary side and only moved their abdomen [34].

### Outcomes

Outcomes were assessed at the beginning and the end of the intervention by the same physicians blinded to group allocation.

#### Primary outcome

*Balance assessment*. The choice of the balance tests depends on the objective of the assessment. Searching the literature, we found that the most commonly used tests are: the Berg Balance Scale (BBS), Timed Up and Go (TUG), and single-leg stance test (SLS) [35]. Often Activities Balance Confidence scale (ABC) is considered as a part of the clinical balance assessment.

*Berg Balance Scale (BBS)*. The BBS is the most widely accepted and psychometrically robust clinical measure of balance for COPD patients [36]. It measures 14 different tasks based on activities such as transfers, reaching, flipping, and single-leg; each attitude was scored on a scale from 0 (unable/unsafe) to 4 (independent/effective/safe), with higher total scores indicating better balance control and a total score out of 56. The threshold was 46, and scores <46 identifies a risk of falling [37].

#### Secondary outcomes

*Timed Up & Go (TUG)*. The TUG as a valid test in COPD, provides a timed measure of balance and functional mobility in our subjects [38]. The test requires the patient to stand up from a standard armchair, walk 3-m at a comfortable pace, return to the chair, and sit down. The test was repeated once more, and the outcome was recorded. Time >16 s was considered to identify a high risk of falling [37].

*Single leg stance test (SLS)*. The SLS test is a static balance test that records the time a participant can support the test on one leg without assistance. The SLS was performed three times with a pause between repetitions, and the best value was recorded [39]. The SLS test is a valid test in COPD that can indicate fall risk in COPD [40]. Values <30 s with open eyes indicated impaired balance in the general population > 60 years old [35]. Values >45 s indicated better balance [37].

*Activities-specific Balance Confidence (ABC) Scale*. The ABC scale requires patients to indicate confidence in performing 16 activities without losing their balance or becoming unstable on an 11-point scale (0% to 100%) [41]. Each element describes a specific activity that requires progressively increased balance control. The highest scores indicate greater confidence in balance or less fear of falling.

*Maximal inspiratory pressure*. The maximal inspiratory pressure (PI_max_) that a subject can produce in the mouth is a simple indicator to assess the strength of the inspiratory muscles [42]. Measurement is made from the residual volume for the maximal inspiratory pressure (PI_max_), using a portable pressure gauge device (MicroRPM, MicroMedical Ltd, Kent, United Kingdom) following the ERS recommendations [42]. The assessments were repeated at least five times (30 s recovery between attempts) and continued until at least one reproducibility value is obtained from the three best measures (in a difference of 10 cmH_2_O between measures) [43].

*Six-minute walk test (6MWT)*. The 6MWT is a valid test that quantifies the functional exercise performance in terms of distance traveled in 6-min in patients with COPD [44]. The test was carried out on a straight course of 40-m in a closed hospital corridor according to the protocol described by the ERS / ATS [44]. Values < 300 are an important risk factor for balance impairment in COPD patients [45].

### Statistical analyses

Sample size calculation was conducted using the two-sample “t-test” for comparing mean differences by the free Web statistics tool BiostaTGV. Assuming a common standard deviation (SD) of 4.7 points in the change in BBS between pre and post-intervention measurements in control (with a change of 1.6 points) and intervention groups (with a change of 7 points) that were reported by Beauchamp et al. [29], with a risk for type one error (α) <5% and risk for type two error (β) of 80%. We determined a sample size of 24 (12 for each group) to detect a between-group difference in balance assessments. In addition, we determined a sample size of 32 (16 for each group) when it was calculated with a risk for type two error (β) of 90%. Three subjects were added in case of loss of follow-up.

Descriptive statistics are expressed as mean ± SD. All Statistical analyses were conducted using SPSS software for Windows, version 20 (IBM Corp., Armonk, NY, USA). Normality distribution was verified using the “Shapiro-Wilk” test. When variables obey the normal distribution, data were compared using parametric t-tests for paired samples and unpaired samples, within and between groups, respectively. Otherwise, data were compared using the non-parametric “Wilcoxon” test for paired samples and the non-parametric “Mann-Whitney” test for unpaired samples, within and between groups, respectively. A chi-squared test was used to compare between Gold stages percentages. The significance limit was set at P < 0.05. Effect size (Cohen’s *d*) was calculated using data at baseline measure and post-training. Values for Cohen’s *d* of 0.2, 0.5, and 0.8 were interpreted as small, moderated, and large, respectively. Pearson’s correlation coefficient was used to analyze the correlation between the change of inspiratory muscle strength and the change of balance parameters for each group and all subjects together.

## Results

**Fig 1:**
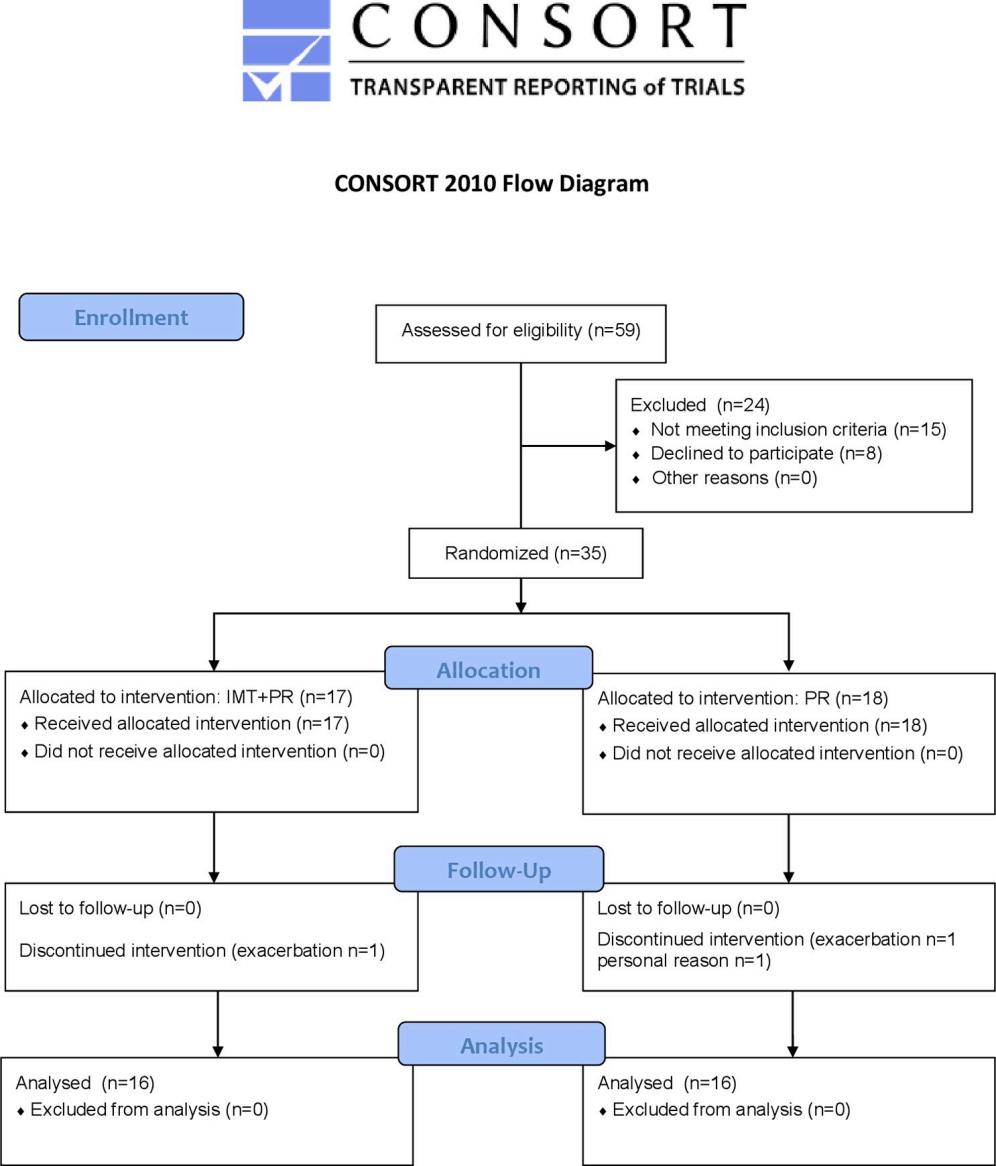
CONSORT (Consolidated Standards of Reporting Trials) 2010 flow diagram. ET: Endurance training; IMT: inspiratory muscle training.

Between January 2019 and September 2019, 59 moderate to very severe COPD patients attended the pulmonary rehabilitation center. Thirty-five male patients were included (Fig 1). Two patients had left the experiments because of exacerbation and one because of personal reasons. The latest two patients were in the control group (Fig 1).

### Baseline data

Baseline patients’ characteristics and values of initial tests are detailed in Table 2. No significant difference was observed between groups at the baseline data.

**Table 2:** Baseline patients’ characteristics and values of initial assessments.

	Experimental group	Control group	P-value
	(n = 16)	(n = 16)	
**Age (years)**	62 ± 5	63 ± 4	0.761
**Height (m)**	1.70 ± 0.05	1.70 ± 0.06	0.922
**Body weight (kg)**	66 ± 9	68 ± 14	0.829
**BMI (kg/m^2^)**	23.13 ± 4.37	23.41 ± 5.19	0.870
**FEV1 (% pred)**	37 ± 7	39 ± 9	0.654
**FEV1/FVC (% pred)**	47 ± 6	44 ± 7	0.441
**GOLD 2 (n)**	1 _(33.3%)_	2 _(66.7%)_	0.560
**GOLD 3 (n)**	11 _(52.4%)_	10 _(47.6%)_	0.721
**GOLD 4 (n)**	4 _(50%)_	4 _(50%)_	1.000
**GOLD Stages (%)**	50	50	0.827
**BBS (point)**	47.7 ± 2.7	47.2 ± 4.5	0.838
**ABC (%)**	75.7 ± 10.0	75.1 ± 12.5	0.724
**TUG (s)**	9.35 ± 1.36	9.28 ± 1.10	0.881
**SLS (s)**	38.88 ± 23.08	38.04 ± 27.14	0.925
**PI_max_ (cmH_2_O)**	62 ± 22	65 ± 22	0.694
**6MWT (m)**	426.3 ± 90.7	428.2 ± 128.7	0.590

Experimental group = IMT+ET. Control group = ET. FEV1/FVC = Forced expiratory volume in 1 second/forced vital capacity. FEV1 = Forced expiratory volume in 1 second. GOLD 2 = Moderate. GOLD 3 = Severe. GOLD 4 = Very severe. PI_max_ = maximal inspiratory pressure. BBS = Berg balance scale. ABC = The activities-specific balance confidence. TUG = Timed up and go test. SLS = Single leg stance test. 6MWT = Six-minute walk test. P < 0.05.

#### Balance

After the training period, functional balance measured with BBS and ABC scales significantly improved between-groups in BBS with a change of 2.7 points (p = 0.019; 95%CI (Confidence Interval) of change: 2.56–3.87 points; 95%CI of performance: 48.07–50.14 points) and in ABC with a change of 7.2% (p = 0.014; 95%CI of change: 6.96–10.53%; 95%CI of performance: 76.75–82.82%). However, there were no significant differences between-groups in TUG (p = 0.18; 95%CI of change: -0.77- -0.40 s; 95%CI of performance: 8.71–9.33 s), and SLS (p = 0.36; 95%CI of change: 1.73–7.51 s; 95%CI of performance: 34.38–47.17 s) (Table 3).

**Table 3:** Pre and post-training assessments.

	Experimental group			Control group			EXP vs. CON	
	(n = 16)			(n = 16)				
Outcomes	Pre-training	Post-training	Change	Pre-training	Post-training	Change	*P*-value	Cohen’s (d)
**BBS (point)**	47.7 ± 2.7	52.3±2.5*	4.6±1.5	47.2 ± 4.5	49.1±4.6*	1.9±0.7	0.019*†*	0.90
**ABC (%)**	75.7 ± 10.0	88.0±9.5*	12.4±3.3	75.1 ± 12.5	80.3±12.5*	5.1±3.4	0.014*†*	0.70
**TUG (s)**	9.35 ± 1.36	8.44±1.36*	-0.91±0.48	9.28 ± 1.10	9.01±1.01*	-0.27±0.29	0.189	-0.48
**SLS (s)**	38.9 ± 23.1	46.6±25.6*	7.7±5.9	38.0 ± 27.1	39.6±27.8	1.5±8.8	0.361	0.26
**PI_max_ (cmH_2_O)**	61.9 ± 21.8	84.8±23.1*	22.9±5.8	64.9 ± 21.7	66.6±22.0	1.7±1.6	0.030*†*	0.81
**6MWT (m)**	426.3 ± 90.7	468.6 ± 98.2*	42.6±25.4	428.2 ± 128.7	457.9 ± 130.6*	29.8±15.2	0.926	0.09

EXPerimental group = IMT +ET. CONtrol group = ET. BBS = Berg balance scale. ABC = The activities-specific balance confidence. TUG = Timed up and go test. SLS = Single leg stance test. PImax = maximal inspiratory pressure. 6MWT = Six-minute walk test.

* Significantly different from the baseline within-group (p<0.001).

† Significantly different post-training between-groups (p<0.05).

Besides, functional balance significantly improved within-group in the experimental group by 10% in BBS (p = 0.001) and 16% in ABC (p = 0.001), and within-group in the control group by 4% in BBS (p = 0.001) and 7% in ABC (p = 0.001). There were significant differences within-group in the experimental group by -10% in TUG (p = 0.001) and 20% in SLS (p = 0.001). There was a significant difference within-group in the control group by -3% (p = 0.001) in the TUG. However, no significant difference was observed within-group in the SLS test for the control group (Table 3).

#### Inspiratory muscle function

After the training period, inspiratory muscle strength (PI_max_) significantly increased only in the experimental group by 37% (p = 0.001; 95%CI: 33.23–49.37%). There was a significant difference between groups with a change of 21.3 cmH_2_O (p = 0.030; 95%CI of change: 8.14–16.48 cmH_2_O; 95% CI of performance: 63.70–75.42 cmH_2_O) (Table 3).

#### The six-minute walk test

There was no significant difference between groups in 6MWT (p = 0.92; 95%CI of change: 29.81–42.18 m; 95%CI of performance: 417.25–473.29 m) (Table 3).

### Correlations between Δ PImax and Δ balance assessments

Significant moderate correlation was observed in the experimental group between PI_max_ and BBS (IMT+ET: r = 0.624, p = 0.010) and between PI_max_ and ABC (IMT+ET: r = 0.550, p = 0.027) (Table 4). However, a significant moderate correlation was only observed in the control group between PI_max_ and BBS (ET r = 0.550, P = 0.027) (Table 4).

**Table 4:** Correlation analysis between inspiratory muscle strength and balance assessments.

		Δ BBS (point)	Δ ABC (%)	Δ TUG (s)	Δ SLS (s)	Δ 6MWT (m)
**Δ PI max (cmH_2_O) Experimental and control groups (n = 32)**	**r**	0.812**	0.777**	-0.550**	0.398*	0.469**
	***p*-value**	0.001	0.001	0.001	0.024	0.007
**Δ PI max (cmH_2_O) Experimental(n = 16)**	**r**	0.624**	0.550*	0.239	-0.097	0.481
	***p*-value**	0.010	0.027	0.373	0.720	0.059
**Δ PI max (cmH_2_O) Control (n = 16)**	**r**	0.550*	0.304	-0.181	0.144	0.365
	***p*-value**	0.027	0.252	0.505	0.595	0.164

Experimental group = IMT +ET. Control group = ET. Δ = Difference between pre and post-training. PI_max_ = maximal inspiratory pressure. BBS = Berg balance scale. ABC = The activities-specific balance confidence. TUG = Timed up and go test. SLS = Single leg stance test. 6MWT = Six-minute walk test.

* Significant correlation (p<0.05) (2-tailed).

** Significant correlation (p<0.01) (2-tailed).

## Discussion

To our knowledge, this study is the first to investigate the effects of additional IMT to ET on balance in COPD patients. As expected, our finding supports the study hypothesis that eight weeks of a specific IMT program combined with ET enhances functional balance, as evidence by improvements in BBS and ABC scales.

### Effects of the training programs on balance

The berg balance scale (BBS) score improved significantly following the addition of IMT to ET by 2.7 points as well as the activities-specific balance confidence (ABC) scale by 7.2%. Besides, we observed significant moderate correlations between inspiratory muscle strength and BBS for both groups as well as ABC but only in the experimental group. This improvement of BBS is in agreement with Beauchamp et al. [28, 29] who found significant improvement by 2.8 points after six weeks of pulmonary rehabilitation (PR) in the same group and after six weeks of balance training added to PR between the experimental group (PR + balance training) and control group (PR only). However, they did not report any significant difference in ABC scale. Despite this absence of significant difference, our findings are in agreement with Mkacher et al. [30] who found significant improvement in ABC by 17.9% and as well in BBS by 6.5 points after six months of balance training added to PR between the experimental group (PR + balance training) and control group (PR only).

Our findings, which demonstrate significant improvement in the functional balance according to BBS and ABC in the favor of the experimental group (IMT+ET), could be explained by potential physiological mechanism(s), as it was described by Ferraro et al. [27], by which IMT added to ET improves balance.

The first potential mechanism is based on the stimulation of the diaphragm and intercostal muscles to respond to a different movement in different tasks and at a different frequency, presumably to support balance during rapid and destabilizing movements of the upper body. Hodges and Gandevia, [18] and Hodges et al. [46] provides definitive evidence that the human diaphragm is involved in the control of postural stability through sudden voluntary movement of the limbs, even though when respiratory demand increases and leads to reduced postural activity of the diaphragm [47]. Besides of diaphragm, intercostal muscles are also shown to be involved in postural control and not only in breathing by supporting balance during rotational movement of the thorax [17, 19].

The second potential mechanism is based on the intra-abdominal pressure. It is known that this pressure increases as the vertical load on the body increases during walking or running [20]. This high intra-abdominal pressure can prolong the lumbar spine and help control the orientation of the spine [20]. Hodges and Gandevia, [21] showed the coactivation of the diaphragm and abdominal muscles to sustain the increase in the intra-abdominal pressure during postural/trunk movement. Therefore, the diaphragm is likely to contribute to postural control of the trunk by elevating intra-abdominal pressure [20].

Our findings, based on these potential physiological mechanisms, support the idea that improvement of inspiratory muscle strength leads to improvements in functional balance, and may help to recover balance which appears to be compromised by increases in trunk muscle activity [24].

However, we did not observe any significant differences between groups in timed up and go (TUG) and in single-leg stance (SLS).

Results of the TUG test are in agreement with Marques et al. [31], who found a significant difference in TUG (8.9 s to 7.2 s) in the same group before and after 12 weeks of PR including balance training in COPD. We are in agreement with Beauchamp et al. [28], who showed a small significant difference in TUG (15.7 s to 14.2 s) in the same group before and after six weeks of PR in COPD. McMeeken et al. [48] showed a significant effect on TUG post knee extensor and flexor muscle strengthening; in our study, the absence of lower limb strengthening could explain the non-significant difference between groups.

Results of the SLS test are contrary to Mkacher et al. [30], who showed significant differences after six months of PR including balance training in COPD between the experimental group (PR + balance training) and the control group (PR only). The same argument as for the TUG test [48], which is the absence of the lower limb strengthening in our training programs, could explain the non-significant difference between groups.

### Effects of the training programs on inspiratory muscle function

Results of this investigation showed that patients improved significantly inspiratory muscle strength (PI_max_) following the addition of the IMT, which supports the previous study in COPD patients [26]. In this study, PI_max_ increased by 21.3 cmH_2_O. This improvement was greater by 3.3 cmH_2_O than those reported by Langer et al. [32], in which they found improvement by 18 cmH_2_O after eight weeks of IMT 7/wk with 60% of PI_max_. Our choice of incremental IMT intensity during eight weeks could explain this difference according to Ambrosino, [33] recommendation, and by giving instructions to encourage active diaphragmatic recruitment during IMT based on the study of Ramsook et al. [34] which demonstrates that simple diaphragmatic breathing instructions can significantly increase the recruitment of the diaphragm during IMT compared to an IMT without any instructions.

This improvement in inspiratory muscle strength could be explained by the adaptive structural changes in inspiratory muscles. Ramirez-Sarmiento et al. [49] found that improvement of strength and endurance after IMT in COPD patients was associated with an increase in the proportion of fibers type *Ⅰ* and with an increase in fibers size type *Ⅱ* in the external intercostal muscles.

### Effects of training programs on the six-minute walk test

The addition of IMT to the ET did not improve significantly the 6MWT between groups, which means that our intervention had the same effect on the functional exercise ability of our patients. Our result is in agreement with the study of Beaumont et al. [50], in which they showed a non-significant difference in the 6MWT between the experimental group (IMT+PR) and the control group (only PR) after 4-week of IMT during pulmonary rehabilitation (PR) program. However, after eight weeks of intervention, we did observe a significant difference within-group for both, experimental (IMT+ET) and control (ET) groups. This improvement in endurance walking within-group for both groups could be explained by the study of Casaburi et al. [51], which showed reductions in exercise lactic acidosis and ventilation as a result of an 8-week endurance exercise training on a cycle ergometer in patients with COPD.

This study had some limitations. First, the absence of female patients due to the higher risk of COPD in Tunisian men as well as the differences in smoking behavior between men and women [52]. Secondly, the training program was performed for two months only. Therefore, further analyses are needed to determine the long-term effect of adding specific inspiratory muscle training to endurance training. Third, COPD patients were not represented in the same proportions according to their gold stages in our study. We tried to recruit more patients from stage Ⅱ or Ⅳ. Therefore, according to the Tunisian population, gold stage Ⅲ was the most found, eligible, and available to participate in our study in comparison with Gold stage Ⅱ and Ⅳ during the rehabilitation period.

## Conclusions

Inspiratory muscle training associated with whole-body endurance training significantly improves functional balance according to BBS and ABC scales in COPD patients. Thus, our findings suggest that inspiratory muscle training combined with endurance training is more effective in improving inspiratory muscle function and generating a better balance control than endurance training alone. Inspiratory muscle training could take part in pulmonary rehabilitation programs aimed at improving balance in patients with COPD.

## Supporting information

S1 Checklist CONSORT checklist of the manuscript. https://doi.org/10.6084/m9.figshare.16616872.
(DOC)

S1 File The protocol of the study in English and French language. https://doi.org/10.6084/m9.figshare.14748567.
(DOCX)

S1 Dataset The individual data points of all subjects’ pre and post training. https://doi.org/10.6084/m9.figshare.16606721.
(XLSX)
