# Supplementary material for: Effects of specific inspiratory muscle training combined with whole-body endurance training program on balance in COPD patients: Randomized controlled trial
Source: PLoS One. 2021 Sep 23;16(9):e0257595. doi: 10.1371/journal.pone.0257595 (PMC8460029; doi:10.1371/journal.pone.0257595)
Supplement: S1 File — https://doi.org/10.6084/m9.figshare.14748567. (DOCX) [file pone.0257595.s002.docx]

# Effects of inspiratory muscle training combined with endurance training on balance in COPD

# Introduction

COPD is a frequent chronic disease, with a significant burden in terms of morbidity and mortality costs. According to the latest estimates from the World Health Organization (WHO) ([2017](http://www.who.int/respiratory/copd/fr/)), 64 million people have COPD, and 3 million people have died from it. Moreover, in industrialized countries, COPD will be the fourth most frequent cause of death in 2030 after being fifth in 2002, with a percentage of mortality worldwide of 7.8 (Mathers and Loncar, [2006](https://www.ncbi.nlm.nih.gov/pubmed/?term=Mathers+C.D.%2C+Loncar+D.+Projections+of+global+mortality+and+burden+of+disease+from+2002+to+2030+PLoS+Med+2006+%3B++3+%3A+e442)). This increase in mortality is mainly explained by the increased epidemic of smoking, the reduction in mortality from other common causes of death (e.g. ischemic heart disease, infectious diseases), and the aging of the world's population (GICOPD, [2016](http://goldcopd.org/global-strategy-diagnosis-management-prevention-copd-2016/)).

In France, 16,000 people die each year from chronic obstructive pulmonary disease (HAS, [2014a](https://www.has-sante.fr/portail/jcms/c_1752117/fr/bronchopneumopathie-chronique-obstructive-bpco-la-has-developpe-des-outils-de-prise-en-charge)). It is estimated at 7.5% in a population over 40, or 3.5 million people. The incidence appears to stabilize in men and increase in women. In 2009, 40,763 people were in ALD (long-term illness) for chronic bronchitis without details. In 2006, crude death rates from COPD were 41/100000 in men and 17/100000 in women aged 45 and over (HAS, [2014b](https://www.has-sante.fr/portail/upload/docs/application/pdf/2012-04/guide_parcours_de_soins_bpco_finale.pdf); [2016a](https://www.has-sante.fr/portail/jcms/c_1518063/fr/bronchopneumopathie-chronique-obstructive)). In Tunisia, Daldoul et al. ([2015](http://www.em-consulte.com/rmr/article/947611)) showed a high prevalence of COPD of 7.8% in Tunisians over 40 years old.

On the other hand, this disease is characterized by a progressive and persistent limitation of airflow and a decrease in parenchymal elasticity (GICOPD, [2016](http://goldcopd.org/global-strategy-diagnosis-management-prevention-copd-2016/)). Consequently, the respiratory muscles remain in contraction for prolonged periods to meet the increased demand for ventilatory flow, which increases the load on the respiratory muscles (Paulin et al., [2003](http://www.scielo.br/scielo.php?pid=S0102-35862003000500007&script=sci_arttext); Montaldo et al., [2000](https://www.ncbi.nlm.nih.gov/pubmed/?term=Chest.+2000%3B117(1)%3A205%E2%80%93225)). Weakness and deconditioning of respiratory and peripheral muscles are assessed in these patients as additional factors used in reduced exercise capacity as well as the quality of life (Hamilton et al., [1995](https://www.ncbi.nlm.nih.gov/pubmed/?term=Am+J+Respir+Crit+Care+Med.+1995%3B152(6+pt+1)%3A+2021%E2%80%932031.)). Inspiratory muscle function is commonly seen in observed patients with COPD (Rochester, [1984](https://www.ncbi.nlm.nih.gov/pubmed/6373180)) who are impaired (decreased strength and/or endurance). Inspiratory muscle dysfunction appears to be the result of geometric changes in the thorax, systemic factors, and/or potential structural changes in the inspiratory muscles (ATS / ERS, [2014](https://www.ncbi.nlm.nih.gov/pubmed/24787074); Arora & Rochester, [1987](https://www.ncbi.nlm.nih.gov/pubmed/3568775)). Inspiratory muscle dysfunction is likely not to limit minimum ventilatory requirements at rest but appears to contribute to dyspnea, decreased exercise capacity, and ventilatory failure during exacerbations (Bégin and Grassino, [1991](https://www.ncbi.nlm.nih.gov/pubmed/2024841)).

Besides, inspiratory muscle training is an effective treatment modality in patients with COPD to improve the strength and endurance of the respiratory muscles, resulting in improved dyspnea, functional exercise, and quality of life (GICOPD, [2016](http://goldcopd.org/global-strategy-diagnosis-management-prevention-copd-2016/)). The American Respiratory Society / European Respiratory Society has required that inspiratory muscle training be primarily applied to stable patients with COPD with suspected or specific inspiratory muscle weakness (ATS / ERS, [2013](https://www.ncbi.nlm.nih.gov/pubmed/?term=Am+J+Respir+Crit+Care+Med.+2013%3B188(8)%3Ae13%E2%80%93e64)). Weakness of the diaphragm and accessory inspiratory muscles is universally found in patients with long-lasting COPD (Polkey et al., [1996](https://www.ncbi.nlm.nih.gov/pubmed/?term=Am+J+Respir+Crit+Care+Med.+1996%3B154(5)%3A1310%E2%80%931317.)). Weak inspiratory muscles lead to dyspnea (Killian and Jones, [1988](https://www.ncbi.nlm.nih.gov/pubmed/?term=Clin+Chest+Med.+1988%3B9(2)%3A237%E2%80%93248.)) and decreased exercise tolerance (Hamilton et al., [1995](https://www.ncbi.nlm.nih.gov/pubmed/?term=Am+J+Respir+Crit+Care+Med.+1995%3B152(6+pt+1)%3A+2021%E2%80%932031.)), as well as hypoxemia, in patients with COPD (Heijidra et al. ., [1995](https://www.ncbi.nlm.nih.gov/pubmed/?term=Thorax.+1995%3B50(6)%3A610%E2%80%93612)). Therefore, inspiratory muscle training is believed to increase inspiratory muscle strength and endurance, decrease dyspnea, as well as improve exercise capacity and quality of life (Gosselink et al., [2011](https://www.ncbi.nlm.nih.gov/pubmed/?term=Eur+Respir+J.+2011%3B37(2)%3A416%E2%80%93425)).

This inspiratory muscle training is defined as the continuous training of inspiratory muscle strength using a specific inspiratory muscle training device (ATS / ERS, [2013](https://www.ncbi.nlm.nih.gov/pubmed/?term=Am+J+Respir+Crit+Care+Med.+2013%3B188(8)%3Ae13%E2%80%93e64)). Inspiratory muscle training has also been applied frequently and has been extended in recent years in patients with COPD (Gosselink et al., [2011](https://www.ncbi.nlm.nih.gov/pubmed/?term=Eur+Respir+J.+2011%3B37(2)%3A416%E2%80%93425)). From the meta-analyzes on patients with COPD, we can conclude that inspiratory muscle training as the only standard therapy improves inspiratory muscle function (strength/endurance), decreases symptoms of dyspnea, and improves exercise capacity (Gosselink et al., [2011](https://www.ncbi.nlm.nih.gov/pubmed/?term=Eur+Respir+J.+2011%3B37(2)%3A416%E2%80%93425); Geddes et al., [2008](https://www.ncbi.nlm.nih.gov/pubmed/?term=Respir+Med+2008%3B102%3A1715%E2%80%9329.)). However, the value of inspiratory muscle training as an adjunct to a general training program is still under debate (Ambrosino, [2011](https://www.ncbi.nlm.nih.gov/pubmed/21282807); McConnell, [2012](https://www.ncbi.nlm.nih.gov/pubmed/?term=J+Physiol+2012%3B590%3A3397%E2%80%938.); Patel et al., [2012](https://www.ncbi.nlm.nih.gov/pubmed/?term=J+Physiol+2012%3B590%3A3393%E2%80%935.); Polkey et al., [2011](https://www.ncbi.nlm.nih.gov/pubmed/?term=Against+Eur+Respir+J+2011%3B37%3A236%E2%80%937.)).

Furthermore, it was concluded that significant additional effects of inspiratory muscle training on more clinically relevant outcomes are likely to be found in patients with inspiratory muscle weakness (ATS / ERS, [2006](https://www.ncbi.nlm.nih.gov/pubmed/?term=Am+J+Respir+Crit+Care+Med+2006%3B173%3A1390%E2%80%93413.)). This was previously defined as a maximum inspiratory pressure (PImax) of less than 60 cm H2O (Gosselink et al., [2011](https://www.ncbi.nlm.nih.gov/pubmed/?term=Eur+Respir+J.+2011%3B37(2)%3A416%E2%80%93425); Beaumont et al., [2015](https://www.ncbi.nlm.nih.gov/pubmed/26170421)). Therefore, it was recommended that future studies in patients with COPD focus specifically on patients with pronounced inspiratory muscle weakness (Gosselink et al., [2011](https://www.ncbi.nlm.nih.gov/pubmed/?term=Eur+Respir+J.+2011%3B37(2)%3A416%E2%80%93425); Decramer, [2009](https://www.ncbi.nlm.nih.gov/pubmed/19342436)).

Recently, it has been shown that integrating inspiratory muscle training into a 4-week rehabilitation program after successful weaning, in patients with COPD, significantly improves functional exercise capacity, and that the addition of inspiratory muscle training, significantly increases functional exercise capacity and increases respiratory muscle strength and power (Dellweg et al., [2017](https://www.ncbi.nlm.nih.gov/pubmed/28137487)). Additionally, after 8 weeks of inspiratory muscle training, inspiratory muscle strength, 6-minute walk test, dyspnea, and quality of life were improved (Chuang et al., [2017](https://www.ncbi.nlm.nih.gov/pubmed/28382660)).

On the other hand, diaphragm movement during standing breathing is greater and faster in patients with COPD than in healthy subjects (Yamada et al., [2017](https://www.ncbi.nlm.nih.gov/pubmed/28065378)). Although the diaphragm is the primary inspiratory muscle (Poole et al., [1997](https://www.ncbi.nlm.nih.gov/pubmed/?term=.+Med+Sci+Sports+Exerc+1997%3B29%3A738e54.)), the human diaphragm is also required to perform postural functions (Hodges & Gandevia, [2000](https://www.ncbi.nlm.nih.gov/pubmed/?term=Activation+of+the+human+diaphragm+during+a+repetitive+postural+task.); Rimmer et al., [1995](https://www.ncbi.nlm.nih.gov/pubmed/?term=Interaction+between+postural+and+respiratory+control+of+human+intercostal+muscles.)). Indeed, Janssens et al. ([2013](https://www.ncbi.nlm.nih.gov/pubmed/23469255)) demonstrated for the first time that people with COPD show poor proprioceptive control, especially when inspiratory muscle strength is diminished. That is, people with COPD, especially those with inspiratory muscle weakness, increased their dependence on proprioceptive signals from the ankle muscles and decreased their dependence on proprioceptive signals from the back muscle during monitoring balance, leading to a decrease in postural stability Janssens et al. ([2013](https://www.ncbi.nlm.nih.gov/pubmed/23469255)). They concluded that these proprioceptive changes might be due to an altered postural contribution of inspiratory muscles to trunk stability. They speculated that more research is needed to determine whether interventions such as proprioceptive training and inspiratory muscle training improve postural balance and reduce the risk of falls in COPD. Besides, Smith et al. ([2016](https://www.ncbi.nlm.nih.gov/pubmed/26471324)) showed that severe COPD is associated with a reduced ability to regain balance and high trunk muscle activity during postural challenges. This increased trunk muscle activity may limit the contribution of trunk movement to balance recovery and may increase the risk of falls.

Moreover, the decreases in exercise capacity, functional mobility, and peripheral muscle performance have been well demonstrated in patients with COPD (Menard et al., [1997](https://www.ncbi.nlm.nih.gov/pubmed/?term=J+Cardiopulm+Rehabil+1997%3B17%3A85-91.); ATS / ERS, [1999](https://www.ncbi.nlm.nih.gov/pubmed/?term=Am+J+Respir+Crit+Care+Med+1999%3B+159(4+pt+2)%3AS1-40.); Maltais et al., [2000](https://www.ncbi.nlm.nih.gov/pubmed/11194778)). However, emerging data indicate that these patients have significant deficits in balance control which may be associated with a high risk of falls in these patients (Butcher et al., [2004](https://www.ncbi.nlm.nih.gov/pubmed/?term=Reductions+in+Functional+Balance%2C+Coordination%2C+and+Mobility+Measures+Among+Patients+With+Stable+Chronic+Obstructive+Pulmonary+Disease); Smith et al., [2010](https://www.ncbi.nlm.nih.gov/pubmed/20206529); Beauchamp et al., [2010](https://www.ncbi.nlm.nih.gov/pubmed/22958342); Beauchamp et al., [2009](https://www.ncbi.nlm.nih.gov/pubmed/19592229)). Indeed, a systematic review aimed to assess whether altered postural controls were more frequent in COPD patients than in age-matched healthy subjects, and to assess the main characteristics of these patients, which contribute to impaired postural control, concluded that: to begin with, impaired postural control is more common in patients with COPD than in healthy subjects of the same age. Second, impaired postural control is associated with loss of muscle strength. Third, impaired postural control is associated with lower functional capacity, independence, and lack of physical activity (Porto et al., [2015](https://www.ncbi.nlm.nih.gov/pubmed/26170652)). More recently, Porto et al. ([2017](https://www.ncbi.nlm.nih.gov/pubmed/28225476)) compared posture control in COPD (n = 93) and healthy subjects (n = 39) during dynamic and static activities. They showed that patients with greater impairment of body balance had a higher incidence of falls, and body balance was more affected with age in patients with COPD than in healthy people.

The ability to maintain stability and balance is essential for functional independence in activities of daily living, mobility, and for preventing falls. Impaired balance has been associated with an increased risk of falls resulting in a higher death rate in the elderly (AGS / BGS / AAO, [2001](https://www.ncbi.nlm.nih.gov/pubmed/11380764)). Falls are a major health problem with serious consequences for older people. It has been estimated that 30-50% of people over 65 years fall at least once a year (Aspray et al., [2006](https://www.ncbi.nlm.nih.gov/pubmed/16364932)). Tinetti et al. ([1988](https://www.ncbi.nlm.nih.gov/pubmed/3205267)) defined a fall as "an event which results in a person unwittingly resting on the ground or at a lower level, not as a result of a major intrinsic event or overwhelming coincidence". Risk factors for falls can be divided into intrinsic and extrinsic. Intrinsic factors are patient-related and include chronic disease, advanced age, gait deviations, muscle weakness, multiple medications, and altered mental status. Extrinsic factors include hazardous activities such as walking on slippery surfaces, inappropriate footwear, unstable living conditions, or environmental hazards (AGS / BGS / AAO, [2001](https://www.ncbi.nlm.nih.gov/pubmed/11380764)).

It is reasonable to assume that the risk of falling increases as the number of risk factors accumulates. The pathophysiological features of COPD suggest that people with this disease have many risk factors that have also been identified in the elderly, such as muscle weakness, multiple medications, polyneuropathy (Ozge et al., [2001](https://www.ncbi.nlm.nih.gov/pubmed/11402511)). Falls are not only associated with mortality and morbidity but are linked to lower overall functioning and early admission to long-term care facilities. Therefore, reducing the risk of falls is an important public health goal (Brown, [1999](http://www.tandfonline.com/doi/abs/10.1080/095939899307775)). Tinetti et al. ([1988](https://www.ncbi.nlm.nih.gov/pubmed/3205267)) showed in older people that the risk of falls was 8% in those who had no risk factors and increased to 78% for those who had four or more risk factors. Falls that do not cause injury frequently start a downward spiral of fear that leads to inactivity and decreased agility, strength, and balance and often results in loss of independence in activities of normal self-care (Tinetti et al., [1994](https://www.ncbi.nlm.nih.gov/pubmed/8169336)).

On the other hand, body balance is a complex functional process that involves not only the somatosensory system but also the vestibular system and visual receptors, with the participation of afferent and efferent structures. Balance can be affected by changes in the sequence of muscle activation, delayed recruitment of synergistic muscles, activation of antagonistic muscles, delayed activation of postural responses, and even by changes in the amplitude of the muscle response. These changes lead to a decrease in the person's ability to detect and control mediolateral and anteroposterior body oscillations statically and during movement (Souchard, [2005](http://www.em-consulte.com/rmr/article/157120); Moxley et al., [1999](https://www.ncbi.nlm.nih.gov/pubmed/10469937?dopt=Abstract)). Current evidence suggests that there is a significant deficit in postural balance in people with COPD and that the pathophysiological basis for these changes is multifactorial (Smith et al., [2010](https://www.ncbi.nlm.nih.gov/pubmed/20206529)). Risk factors for falls in subjects with COPD include lower limb weakness, balance disturbances, nutritional impoverishment, malnutrition, affective states, cognitive impairment, and drug use (Roig et al. ., [2009](https://www.ncbi.nlm.nih.gov/pubmed/19419852?dopt=Abstract)). Several studies suggest that the control of anteroposterior balance is impaired in people with severe COPD who need supplemental oxygen (Roig et al., [2009](https://www.ncbi.nlm.nih.gov/pubmed/19419852?dopt=Abstract); Butcher et al., [2004](https://www.ncbi.nlm.nih.gov/pubmed/15286536?dopt=Abstract)).

Over the years, several instruments have been developed to quantitatively measure balance in the elderly population. These screening instruments are used to assess the ability to maintain balance and subsequently identify individuals who are at significant risk of falling in the near future (Mancini & Horak, [2010](https://www.ncbi.nlm.nih.gov/pubmed/20485226)). The choice of balance tests depends on the purpose of the assessment. By searching the literature, we have found that the most common tests used are Berg's Balance Scale (BBS), Timed Up & Go (TUG), and Single Leg Position Test (SLS). Often, the Activity of balance Confidence Scale (ABC) is considered part of the assessment of clinical workup (Alexander, [1994](https://www.ncbi.nlm.nih.gov/pubmed/8277123); Berg & Norman, [1996](https://www.ncbi.nlm.nih.gov/pubmed/8890112); Bobannon, [2006](http://journals.lww.com/topicsingeriatricrehabilitation/Abstract/2006/01000/Single_Limb_Stance_Times__A_Descriptive.10.aspx); Mancini and Horak, [2010](https://www.ncbi.nlm.nih.gov/pubmed/20485226)). Besides, the 6-minute walk test (6MWT) provides a good estimate of aerobic capacity/endurance and overall functional performance (Bean et al., [2002](https://www.ncbi.nlm.nih.gov/pubmed/12403805)). The measurement properties of this test have been well established in the COPD population (Solway et al., [2001](https://www.ncbi.nlm.nih.gov/pubmed?term=%22Chest%22%5BJournal%5D%20AND%202001%5BPDAT%5D%20AND%20119%5BVOL%5D%20AND%20256-70%5BPAGE%5D); ERS / ATS, [2014](https://www.ncbi.nlm.nih.gov/pubmed/?term=An+official+European+Respiratory+Society%2F+American+Thoracic+Society+technical+standard%3A+field+walking+tests+in+chronic+respiratory+disease)). This test could be associated with the assessment of balance in patients with COPD. To our knowledge, the effects of inspiratory muscle training combined with rehabilitation programs on balance in patients with COPD have not been studied. Experimental approach

All measurements will be taken before and after the training programs. At the respiratory level, we measure maximal inspiratory pressure (PImax) and spirometry measurements. For balance, we will proceed by single-leg position test, timed Up & Go, the ABC questionnaires, and the Berg Balance Scale (Crisan et al., [2015](https://www.ncbi.nlm.nih.gov/pubmed/25768731)). We will use the 6-minute walk us a functional exercise test (CR-10, RPD, heart rate, oxygen saturation, and ventilation will be measured in this test).

We will consider two groups of 12 patients minimum. Group 1 will do inspiratory muscle training combined with endurance training. Group 2 will be a control group and will only perform the endurance training. The training programs will be carried out over eight weeks.

# Subjects

Patients will be admitted to the study if they had COPD diagnosed according to the criteria of the American Thoracic Society / European Respiratory Society (Vestbo et al., [2013](https://www.ncbi.nlm.nih.gov/pubmed/22878278)). They will be divided into 2 groups, an experimental group versus a control group. Only patients with a forced expiratory volume within 1 s (FEV1) <80% predicted and FEV1 / forced vital capacity (FVC) <70%) will be eligible to participate in the study. The exclusion criteria consist of (1) diagnosed psychiatric or cognitive disorders, (2) progressive neurological or neuromuscular disorders, (3) serious orthopedic problems having a major impact on daily activities, and (4) prior inclusion in a rehabilitation program (<1 year) (Charususin et al., [2013](https://www.ncbi.nlm.nih.gov/pubmed/?term=Inspiratory+muscle+training+protocol+for+patients+with+chronic+obstructive+pulmonary+disease+(IMTCO+study)%3A+a+multicentre+randomised+controlled+trial); Beaumont et al., [2018](https://www.ncbi.nlm.nih.gov/pubmed/29371379)).

# Inspiratory muscle training program

Patients will be randomized to inspiratory muscle training. The training will be done once a day for 7 days for 8 weeks. In inspiratory muscle training, we will use a device (PowerBreathe® Medic, IMT Technologies Ltd, Birmingham, UK). The training consists of doing 2 daily sets of 30 breaths between cut off from 5 to 10 minutes of recovery (Langer et al., [2015](https://www.ncbi.nlm.nih.gov/pubmed/?term=Efficacy+of+a+Novel+Method+for+Inspiratory+Muscle+Training+in+People+With+Chronic+Obstructive+Pulmonary+Disease)). With a load of 50% of basal PImax, incremental by 10% of PImax every 2 weeks of training (50%, 60%, 70%, and 80% PI max) (Ambrosino, [2018](https://www.ncbi.nlm.nih.gov/pubmed/29371389)). Patients will be instructed to emphasize the use of their diaphragms and to ensure that their abdomens "come off" during each inspiratory maneuver (Ramsook et al., [2016](https://www.ncbi.nlm.nih.gov/pubmed/?term=Diaphragm+Recruitment+Increases+during+a+Bout+of+Targeted+Inspiratory+Muscle+Training)). They will be familiarized with diaphragmatic breathing by sitting upright in a chair and placing one hand on the abdomen and the other hand on the ribs along the anterior axillary line. Subjects then breathe and attempt to keep their hand on the stationary rib and only move their abdomen (Ramsook et al., [2016](https://www.ncbi.nlm.nih.gov/pubmed/?term=Diaphragm+Recruitment+Increases+during+a+Bout+of+Targeted+Inspiratory+Muscle+Training)). This method has been shown to induce an increase in diaphragm participation during natural breathing resulting in improved functional capacity in COPD during a respiratory training program emphasizing the use of the diaphragm (Yamaquti et al., [2012](https://www.ncbi.nlm.nih.gov/pubmed/22464088)).

# Endurance training program

The endurance-training program will be carried out for 8 weeks, 3 days a week (IMT and control group). The session process will include aerobic exercise on a treadmill (30 min per day) and then upper and lower limb stretching (Beauchamp et al., [2010](https://www.ncbi.nlm.nih.gov/pubmed/20801268)).

# Measures

The following measures will be completed before and after the training programs.

## Maximum respiratory pressures

The maximum inspiratory pressure (PImax) that a subject can produce in the mouth is a simple indict by which to assess the strength of the inspiratory muscles (ATS/ERS, [2002](https://www.ncbi.nlm.nih.gov/pubmed/12186831)). The measurements will be taken from the residual volume for maximum inspiratory pressure (PImax), using a portable Manometer device (MicroRPM, MicroMedical Ltd, Kent, United Kingdom) according to the (ATS/ERS, [2002](https://www.ncbi.nlm.nih.gov/pubmed/12186831)) statement. The evaluations will be repeated at least five times (30 s recovery between attempts), and should be continued until at least reproducibility is obtained from the best three measurements (within a difference of 10 cm H2O between the measurements) (Charususin et al., [2013](https://www.ncbi.nlm.nih.gov/pubmed/?term=Inspiratory+muscle+training+protocol+for+patients+with+chronic+obstructive+pulmonary+disease+(IMTCO+study)%3A+a+multicentre+randomised+controlled+trial)).

## Spirometry test

Respiratory maneuvers will be performed using a spirometer (EsayOne Diagnostic, Switzerland) (Barr et al., [2008](https://www.ncbi.nlm.nih.gov/pubmed/18364054)). The following ventilatory variables will be measured at least three times and only reproducible values ​​will be saved. Respiratory functional explorations will be performed before training programs.

Forced vital capacity (expressed in liters): FVC

It corresponds to the volume of gas exhaled during a full forced exhalation starting from the maximum inspiration position. FVC may be underestimated if the subject is not given sufficient time to empty his lungs at low volume, that is, at the stage where the expiratory flow is determined by the flow restrictor mechanism.

Maximum second expired volume (expressed in liters): FEV1

It is obtained after a maximum inspiration followed by a brief period of apnea. The subject should perform a maximum forced and rapid exhalation. FEV1 depends on the subject's muscular effort and cooperation. This is a very widely used and reproducible parameter.

Tiffeneau ratio = (FEV1 / FVC × 100)

The FEV1 / FVC ratio makes it possible to identify the percentage of the vital capacity that can be mobilized in one second. Obstructive ventilatory defect (OVD) is defined as an FEV1 / FVC ratio of less than 0.7. The severity of OVD is determined by the value of FEV1 expressed as a percentage of the theoretical value (Quanjer et al., [1993](https://www.ncbi.nlm.nih.gov/pubmed/?term=Eur+Respir+J+Suppl.+1993+Mar%3B16%3A85-100)). The reversibility of the respiratory function is checked as follows: 15 minutes after having inspired four puffs of a B2-mimetic (Ventolin), the subject carries out a new measurement: the reversibility is recognized if the change in FEV1 after taking bronchodilator is greater than 12% of the initial value (Meslier et al., [1989](https://www.ncbi.nlm.nih.gov/pubmed/?term=Eur+Respir+J+1989%3B+2%3A497-505.))**.**

## Berg Balance Scale

We chose [BBS](https://www.has-sante.fr/portail/upload/docs/application/pdf/Evaluation_%20fonctionnelle_%20AVC_ref.pdf) (Berg et al., [1992](https://www.ncbi.nlm.nih.gov/pubmed/?term=Can+J+Public+Health+1992%3B83(Suppl+2)%3AS7-11.)) as the primary outcome for this study because it is the most widely accepted and psychometrically robust clinical measure of balance for the elderly (Tyson et Connell, [2009](https://www.ncbi.nlm.nih.gov/pubmed/?term=Clin+Rehabil+2009%3B23%3A824-40.)) and considered the gold standard test for static and dynamic equilibrium capacities (Langley et Mackintosh, [2007](http://nsuworks.nova.edu/ijahsp/vol5/iss4/13/)). Activities such as handoffs, reaching, flipping, and one-legged stance were rated on a scale from 0 (unable/unsecured) to 4 (independent/effective/safe), with scores over high indicating better balance control.

The measurement obtained using the BBS showed internal consistency, within and between groups, reliability, content validity, construct validity, and predictive validity for determining the risk of falls in the elderly (Finchet al., [2002](https://www.ncbi.nlm.nih.gov/nlmcatalog/101175542)). A cutoff score of 46 and less has been identified as a useful score for successfully identifying those at risk for falls (Lajoie et Gallagher, [2004](https://www.ncbi.nlm.nih.gov/pubmed/?term=Arch+Gerontol+Geriatr+2004%3B38%3A11-26.); Shumway et al., [1997](https://www.ncbi.nlm.nih.gov/pubmed/9256869)). A change of 3.3 (or ≥ 4 points) has been suggested to represent a minimal detection change (MDC) in elderly patients with baseline BBS scores of 45 to 56 points. MDC scores for subjects with lower basic BBS range from 5 to 6 points for older people living in a community setting (Donoghue et al., [2009](https://www.ncbi.nlm.nih.gov/pubmed/?term=J+Rehabil+Med+2009%3B41%3A343-6.)).

## Timed Up & Go

We chose the TUG test to provide a timed measure of balance and functional mobility in our patients (Podsiadlo et Richardson, [1991](https://www.ncbi.nlm.nih.gov/pubmed/1991946)). The test requires the patient to stand up from a standard chair, walk 3 m at a comfortable pace, return to the chair, and sit down. A trial will be carried out (unregistered) where individuals will be allowed to use a walking aid if necessary. The TUG has high reliability and within and between-group predictive validity for falls in adults living in community settings (Finchet al., [2002](https://www.ncbi.nlm.nih.gov/nlmcatalog/101175542); Podsiadlo et Richardson, [1991](https://www.ncbi.nlm.nih.gov/pubmed/1991946)). A cut-off score of 16 seconds or more predicted falls in elderly people living in a community residence (Okumiya et al., [1998](https://www.ncbi.nlm.nih.gov/pubmed/9670889)). Reported MDC scores vary from 4 seconds in patients with Alzheimer's disease (Ries et al., [2009](https://www.ncbi.nlm.nih.gov/pubmed/?term=Phys+Ther+2009%3B89%3A569-79.)) to 15 seconds in frail elderly patients (Nordin et al., [2006](https://www.ncbi.nlm.nih.gov/pubmed/?term=Phys+Ther+2006%3B86%3A646-55.)). TUG is a valid and reactive test in COPD (Mesquita et al., [2016](https://www.ncbi.nlm.nih.gov/pubmed/27165963)).

## Single leg position test

The SLS (Single Limb Stance Times) a static balance test that records the time a participant can withstand on one leg without assistance. The SLS will be performed 3 times with a break between repetitions and the best value will be used. The SLS will be established with eyes open. In a descriptive meta-analysis, Bohannon et al. ([2006](http://journals.lww.com/topicsingeriatricrehabilitation/Abstract/2006/01000/Single_Limb_Stance_Times__A_Descriptive.10.aspx)) presented the appropriate standards for SLS.

## The ABC scale

The [ABC](http://www.archives-pmr.org/article/S0003-9993(06)01318-9/pdf) scale requires patients to indicate their confidence in performing 16 activities without losing balance or becoming unstable on an 11 point scale (0% to 100%) (Myers et al., [1998](https://www.ncbi.nlm.nih.gov/pubmed/?term=J+Gerontol+A+Biol+Sci+Med+Sci+1998%3B53%3A+M287-94.)). Each item describes a specific activity that requires progressively increased balance control. Higher results indicate greater confidence in balance or less fear of falling. The ABC scale has good test-retest reliability, internal consistency, and predictive ability for falls in older people residing in a community setting (Finchet al., [2002](https://www.ncbi.nlm.nih.gov/nlmcatalog/101175542); Myers et al., [1998](https://www.ncbi.nlm.nih.gov/pubmed/?term=J+Gerontol+A+Biol+Sci+Med+Sci+1998%3B53%3A+M287-94.)). A 13% change has been shown to reflect a minimal detection change (MDC) for this measurement (Steffen et Seney, [2008](https://www.ncbi.nlm.nih.gov/pubmed/?term=Phys+Ther+2008%3B88%3A733-46.)).

## 6-minute walk test

The 6MWT is a valid, responsive, interpretable, and self-assessed test that quantifies functional exercise capacity in terms of distance traveled in 6 minutes in patients with COPD (Burge et al., [2000](https://www.ncbi.nlm.nih.gov/pubmed/?term=BMJ+2000%3B320%3A1297-303.)). The test will be carried out on a straight course of 40 meters in a closed corridor according to the protocol described by the ERS/ATS ([2014](https://www.ncbi.nlm.nih.gov/pubmed/?term=An+official+European+Respiratory+Society%2F+American+Thoracic+Society+technical+standard%3A+field+walking+tests+in+chronic+respiratory+disease)). During this test, the ventilatory parameters will be evaluated by a Spiropalm (COSMED Spiropalm, Rome, Italy) (Ponomareva et al., [2015](http://erj.ersjournals.com/content/46/suppl_59/PA1029)). During each 6MWT, patients will have standardized instructions and encouragement. Two tests will be carried out to take into account the possible improvements resulting from familiarization, with the greater distance recorded. Each 6MWT will be separated by a minimum of 30 minutes. The measurement properties of this test are well established in the COPD population (Solway et al., [2001](https://www.ncbi.nlm.nih.gov/pubmed?term=%22Chest%22%5BJournal%5D%20AND%202001%5BPDAT%5D%20AND%20119%5BVOL%5D%20AND%20256-70%5BPAGE%5D); ERS/ATS, [2014](https://www.ncbi.nlm.nih.gov/pubmed/?term=An+official+European+Respiratory+Society%2F+American+Thoracic+Society+technical+standard%3A+field+walking+tests+in+chronic+respiratory+disease)). A dyspnea level perception scale (RPD: 0-10) (Wilson et Jones, [1991](https://www.ncbi.nlm.nih.gov/pubmed/?term=Long-term+reproducibility+of+Borg+scale+estimates+of+breathlessness+during+exercise.)) will be used after each test.

***Statistical analyses***

Assuming a common standard deviation (SD) of 4.7 points in the change in BBS between pre and post-intervention measurements in control and intervention groups that were reported by Beauchamp et al. [2013](https://pubmed.ncbi.nlm.nih.gov/23975185/), with risk for type one error (α) <5% and risk for type two error (β) of 80%. We determined a sample size of 24 (12 for each group) to detect a between-group difference in balance assessments. Considering a risk of type 2 error (β) of 90%, we determined a sample size of 32 (16 for each group).

Descriptive statistics are expressed as mean ± SD. All Statistical analyses were conducted using SPSS software for Windows, version 20 (IBM Corp., Armonk, NY, USA). Normality distribution was verified using the “Shapiro-Wilk” test. When variables obey the normal distribution, data were compared using parametric t-tests for paired samples and unpaired samples, within and between groups, respectively. Otherwise, data were compared using the non-parametric “Wilcoxon” test for paired samples and the non-parametric “Mann-Whitney” test for unpaired samples, within and between groups, respectively. A chi-squared test was used to compare between Gold stages percentages. The significance limit was set at P < 0.05. Effect size (Cohen’s *d*) was calculated using data at baseline measure and post-training. Values for Cohen’s *d* of 0.2, 0.5, and 0.8 were interpreted as small, moderated, and large, respectively. Pearson’s correlation coefficient was used to analyze the correlation between the change of inspiratory muscle strength and the change of balance parameters for each group and all subjects together.

**Effets de l’entraînement des muscles inspiratoires combiné à la l’entrainement en endurance sur l’équilibre chez les BPCO**

**Introduction**

La BPCO est une maladie chronique fréquente, avec un poids important en termes de morbidité et de coûts de mortalité. Selon les dernières estimations de l’organisation mondiale de la santé (OMS) ([2017](http://www.who.int/respiratory/copd/fr/)), 64 millions de personnes ont une BPCO, et 3 millions de personnes en sont mortes. D’ailleurs, dans les pays industrialisés, la BPCO figurera au quatrième rang des causes de mortalité les plus fréquentes en 2030 après avoir était au cinquième rang en 2002, avec un pourcentage de mortalité dans le monde de 7,8 (Mathers et Loncar, [2006](https://www.ncbi.nlm.nih.gov/pubmed/?term=Mathers+C.D.%2C+Loncar+D.+Projections+of+global+mortality+and+burden+of+disease+from+2002+to+2030+PLoS+Med+2006+%3B++3+%3A+e442)). Cette augmentation de mortalité s’explique principalement par l’épidémie croissante de tabagisme, la réduction de la mortalité due à d’autres causes communes de décès (par exemple, maladie cardiaque ischémique, maladies infectieuses) et le vieillissement de la population mondiale (GICOPD, [2016](http://goldcopd.org/global-strategy-diagnosis-management-prevention-copd-2016/)).

En France, 16 000 personnes meurent chaque année des suites de la bronchopneumopathie chronique obstructive (HAS, [2014a](https://www.has-sante.fr/portail/jcms/c_1752117/fr/bronchopneumopathie-chronique-obstructive-bpco-la-has-developpe-des-outils-de-prise-en-charge)). Elle est estimée à 7,5 % dans une population de plus de 40 ans, soit 3,5 millions de personnes. L’incidence semble se stabiliser chez l’homme et augmenter chez la femme. En 2009, 40 763 personnes étaient en ALD (affection de longue durée) pour bronchite chronique sans précision. En 2006, les taux bruts de mortalité par BPCO étaient de 41/100 000 chez les hommes et 17/100 000 chez les femmes âgés de 45 ans et plus (HAS, [2014b](https://www.has-sante.fr/portail/upload/docs/application/pdf/2012-04/guide_parcours_de_soins_bpco_finale.pdf) ; [2016a](https://www.has-sante.fr/portail/jcms/c_1518063/fr/bronchopneumopathie-chronique-obstructive)). En Tunisie, Daldoul et al. ([2015](http://www.em-consulte.com/rmr/article/947611)) ont montré une forte prévalence de la BPCO de 7,8% chez les Tunisiens de plus de 40 ans.

D’autre part, cette maladie se caractérise aussi par une limitation progressive et persistante de l’écoulement de l’air et une diminution de l’élasticité parenchymateuse (GICOPD, [2016](http://goldcopd.org/global-strategy-diagnosis-management-prevention-copd-2016/)). Par conséquent, les muscles respiratoires restent en contraction pendant des périodes prolongées dans le but de répondre à la demande accrue d’écoulement ventilatoire, ce qui augmente la charge sur les muscles respiratoires (Paulin et al., [2003](http://www.scielo.br/scielo.php?pid=S0102-35862003000500007&script=sci_arttext) ; Montaldo et al., [2000](https://www.ncbi.nlm.nih.gov/pubmed/?term=Chest.+2000%3B117(1)%3A205%E2%80%93225)). La faiblesse et le déconditionnement des muscles respiratoires et périphériques sont reconnus chez ces patients comme des facteurs supplémentaires impliqués dans la réduction de la capacité d’exercice ainsi dans la qualité de vie (Hamilton et al., [1995](https://www.ncbi.nlm.nih.gov/pubmed/?term=Am+J+Respir+Crit+Care+Med.+1995%3B152(6+pt+1)%3A+2021%E2%80%932031.)). La fonction des muscles inspiratoires est fréquemment constatée chez les patients souffrant de BPCO (Rochester, [1984](https://www.ncbi.nlm.nih.gov/pubmed/6373180)) qui ont des facultés affaiblies (diminution de la force et/ou de l'endurance). Le dysfonctionnement des muscles inspiratoires semble être le résultat de changements géométriques du thorax, des facteurs systémiques et/ou des changements structurels potentiels des muscles inspiratoires (ATS/ERS, [2014](https://www.ncbi.nlm.nih.gov/pubmed/24787074) ; Arora et Rochester, [1987](https://www.ncbi.nlm.nih.gov/pubmed/3568775)). Il est probable que le dysfonctionnement des muscles inspiratoires ne limite pas les besoins ventilatoires minimaux au repos, mais il semble contribuer à la dyspnée, à la diminution de la capacité d'exercice et à l'insuffisance ventilatoire pendant les exacerbations (Bégin et Grassino, [1991](https://www.ncbi.nlm.nih.gov/pubmed/2024841)).

Par ailleurs, l’entraînement des muscles inspiratoires a été montré comme une modalité de traitement efficace chez les patients atteints de BPCO afin d’améliorer la force et l’endurance des muscles respiratoires, ce qui entraîne une amélioration de la dyspnée, de la capacité d’exercice fonctionnelle et de la qualité de vie (GICOPD, [2016](http://goldcopd.org/global-strategy-diagnosis-management-prevention-copd-2016/)). L’American Respiratory Society/European Respiratory Society suggère que l’entraînement des muscles inspiratoires est principalement appliqué aux patients stables atteints de BPCO avec une faiblesse des muscles inspiratoires suspectée ou spécifique (ATS/ERS, [2013](https://www.ncbi.nlm.nih.gov/pubmed/?term=Am+J+Respir+Crit+Care+Med.+2013%3B188(8)%3Ae13%E2%80%93e64)). La faiblesse du diaphragme et des muscles inspiratoires accessoires est universellement trouvé chez les patients atteints de BPCO longue durée (Polkey et al., [1996](https://www.ncbi.nlm.nih.gov/pubmed/?term=Am+J+Respir+Crit+Care+Med.+1996%3B154(5)%3A1310%E2%80%931317.)). La faiblesse des muscles inspiratoires entraîne une dyspnée (Killian et Jones, [1988](https://www.ncbi.nlm.nih.gov/pubmed/?term=Clin+Chest+Med.+1988%3B9(2)%3A237%E2%80%93248.)) et une diminution de la tolérance de l’exercice (Hamilton et al., [1995](https://www.ncbi.nlm.nih.gov/pubmed/?term=Am+J+Respir+Crit+Care+Med.+1995%3B152(6+pt+1)%3A+2021%E2%80%932031.)), ainsi que l’hypoxémie, chez les patients atteints de BPCO (Heijidra et al., [1995](https://www.ncbi.nlm.nih.gov/pubmed/?term=Thorax.+1995%3B50(6)%3A610%E2%80%93612)). Par conséquent, l’entraînement des muscles inspiratoires est censé augmenter la force et l’endurance des muscles inspiratoires, diminue la dyspnée, ainsi que d’améliorer la capacité d’exercice et la qualité de vie (Gosselink et al., [2011](https://www.ncbi.nlm.nih.gov/pubmed/?term=Eur+Respir+J.+2011%3B37(2)%3A416%E2%80%93425)).

Cet entraînement des muscles inspiratoires est défini comme l’entraînement continu de la force des muscles inspiratoires en utilisant un dispositif spécifique de l’entraînement des muscles inspiratoires (ATS/ERS, [2013](https://www.ncbi.nlm.nih.gov/pubmed/?term=Am+J+Respir+Crit+Care+Med.+2013%3B188(8)%3Ae13%E2%80%93e64)). L’entraînement des muscles inspiratoires a également été appliqué fréquemment et a été largement étudiée ces dernières années chez les patients atteints de BPCO (Gosselink et al., [2011](https://www.ncbi.nlm.nih.gov/pubmed/?term=Eur+Respir+J.+2011%3B37(2)%3A416%E2%80%93425)). À partir des méta-analyses sur les patients atteints de BPCO, nous pouvons conclure que l’entraînement des muscles inspiratoires en tant que seule thérapie standard améliore la fonction des muscles inspiratoires (force / endurance), diminue les symptômes de dyspnée et améliore la capacité d’exercice (Gosselink et al., [2011](https://www.ncbi.nlm.nih.gov/pubmed/?term=Eur+Respir+J.+2011%3B37(2)%3A416%E2%80%93425) ; Geddes et al., [2008](https://www.ncbi.nlm.nih.gov/pubmed/?term=Respir+Med+2008%3B102%3A1715%E2%80%9329.)). Cependant, la valeur de l’entraînement des muscles inspiratoires en tant que complément à un programme générale d’entraînement est encore en débat (Ambrosino, [2011](https://www.ncbi.nlm.nih.gov/pubmed/21282807) ; McConnell, [2012](https://www.ncbi.nlm.nih.gov/pubmed/?term=J+Physiol+2012%3B590%3A3397%E2%80%938.) ; Patel et al., [2012](https://www.ncbi.nlm.nih.gov/pubmed/?term=J+Physiol+2012%3B590%3A3393%E2%80%935.) ; Polkey et al., [2011](https://www.ncbi.nlm.nih.gov/pubmed/?term=Against+Eur+Respir+J+2011%3B37%3A236%E2%80%937.)).

Par ailleurs, il a été conclu que des effets supplémentaires significatifs de l’entraînement des muscles inspiratoires sur des résultats plus cliniquement pertinents sont susceptibles d’être trouvés chez des patients avec une faiblesse au niveau des muscles inspiratoire (ATS/ERS, [2006](https://www.ncbi.nlm.nih.gov/pubmed/?term=Am+J+Respir+Crit+Care+Med+2006%3B173%3A1390%E2%80%93413.)). Ceci a été défini précédemment comme une pression inspiratoire maximale (PI_max_) de moins de 60 cm H_2_O (Gosselink et al., [2011](https://www.ncbi.nlm.nih.gov/pubmed/?term=Eur+Respir+J.+2011%3B37(2)%3A416%E2%80%93425) ; Beaumont et al., [2015](https://www.ncbi.nlm.nih.gov/pubmed/26170421)). Par conséquent, il a été recommandé que les futures études chez les patients atteints de BPCO se concentrent spécifiquement sur les patients avec une faiblisse des muscles inspiratoires prononcée (Gosselink et al., [2011](https://www.ncbi.nlm.nih.gov/pubmed/?term=Eur+Respir+J.+2011%3B37(2)%3A416%E2%80%93425) ; Decramer, [2009](https://www.ncbi.nlm.nih.gov/pubmed/19342436)).

Récemment, il a été montré que l’intégration de l’entraînement des muscles inspiratoires dans un programme de rééducation de 4 semaines après un sevrage réussie, chez des patients souffrants de BPCO, améliore significativement la capacité d’exercice fonctionnel, et que l’addition de l’entraînement des muscles inspiratoires, augmente considérablement la capacité d’exercice fonctionnel et augmente la force et la puissance des muscles respiratoires (Dellweg et al., [2017](https://www.ncbi.nlm.nih.gov/pubmed/28137487)). De plus, après 8 semaines d’entraînement des muscles inspiratoires, la force des muscles inspiratoires, le test de marche de 6 minutes, la dyspnée et la qualité de vie ont été amélioré (Chuang et al., [2017](https://www.ncbi.nlm.nih.gov/pubmed/28382660)).

D’autres part, il a été montré que le mouvement du diaphragme pendant la respiration en position debout est plus grand et plus rapide chez les patients atteints de BPCO que chez des sujets normaux (Yamada et al., [2017](https://www.ncbi.nlm.nih.gov/pubmed/28065378)). Bien que le diaphragme soit le principal muscle d’inspiration (Poole et al., [1997](https://www.ncbi.nlm.nih.gov/pubmed/?term=.+Med+Sci+Sports+Exerc+1997%3B29%3A738e54.)), le diaphragme humain est également nécessaire pour exécuter des fonctions posturales (Hodges et Gandevia, [2000](https://www.ncbi.nlm.nih.gov/pubmed/?term=Activation+of+the+human+diaphragm+during+a+repetitive+postural+task.) ; Rimmer et al., [1995](https://www.ncbi.nlm.nih.gov/pubmed/?term=Interaction+between+postural+and+respiratory+control+of+human+intercostal+muscles.)). En effet, Janssens et al. ([2013](https://www.ncbi.nlm.nih.gov/pubmed/23469255)) ont démontré pour la première fois que les personnes atteintes de BPCO montrent un mauvais contrôle proprioceptif, en particulier lorsque la force des muscles inspiratoires est diminuée. C’est-à-dire, les personnes atteintes de BPCO, en particulier celles présentant une faiblesse des muscles inspiratoires, ont augmenté leur dépendance aux signaux proprioceptifs des muscles de la cheville et ont diminué leur dépendance aux signaux proprioceptifs du muscle dorsal pendant le contrôle de l’équilibre, entrainant une diminution de la stabilité posturale (Janssens et al., [2013](https://www.ncbi.nlm.nih.gov/pubmed/23469255)). Ils ont conclu que ces changements proprioceptifs peuvent être dus à une altération de la contribution posturale des muscles inspiratoires à la stabilité du tronc. Ils ont spéculé que des recherches supplémentaires sont nécessaires pour déterminer si des interventions telles que l’entraînement proprioceptif et l’entraînement des muscles inspiratoires améliorent l’équilibre postural et réduisent le risque de chute chez les BPCO. En outre, Smith et al. ([2016](https://www.ncbi.nlm.nih.gov/pubmed/26471324)) ont montré que la BPCO sévère est associée à une capacité réduite à retrouver l’équilibre et à une grande activité musculaire du tronc pendant les défis posturaux. Cette activité musculaire augmenté du tronc peut limiter la contribution des mouvements du tronc à la récupération de l’équilibre et pourrait contribuer à accroître le risque de chutes.

Par ailleurs, la diminution de la capacité d'exercice, de la mobilité fonctionnelle et de la performance musculaire périphérique a été bien démontrée chez les patients atteints de BPCO (Menardet al., [1997](https://www.ncbi.nlm.nih.gov/pubmed/?term=J+Cardiopulm+Rehabil+1997%3B17%3A85-91.) ; ATS/ERS, [1999](https://www.ncbi.nlm.nih.gov/pubmed/?term=Am+J+Respir+Crit+Care+Med+1999%3B+159(4+pt+2)%3AS1-40.) ; Maltais et al., [2000](https://www.ncbi.nlm.nih.gov/pubmed/11194778)). Cependant, des données émergentes montrent que ces patients ont des déficits important dans le contrôle de l’équilibre qui peut être associées à un risque de chute élevé chez ces patients (Butcher et al., [2004](https://www.ncbi.nlm.nih.gov/pubmed/?term=Reductions+in+Functional+Balance%2C+Coordination%2C+and+Mobility+Measures+Among+Patients+With+Stable+Chronic+Obstructive+Pulmonary+Disease) ; Smith et al., [2010](https://www.ncbi.nlm.nih.gov/pubmed/20206529) ; Beauchamp et al., [2010](https://www.ncbi.nlm.nih.gov/pubmed/22958342) ; Beauchamp et al., [2009](https://www.ncbi.nlm.nih.gov/pubmed/19592229)). En effet, Une revue de la littérature avait comme objectifs d’évaluer si les contrôles posturaux altérés étaient plus fréquents chez les patients BPCO que chez les sujets sains appariés selon l’âge et d’évaluer les principales caractéristiques de ces patients qui contribuent à un contrôle posturale altéré, elle a conclu que : Premièrement, l'altération du contrôle postural est plus fréquente chez les patients atteints de BPCO que chez les sujets en bonne santé d'âge égal. Deuxièmement, l'altération du contrôle postural est associée à une perte de force musculaire. Troisièmement, l'altération du contrôle postural est associée à une capacité fonctionnelle, à une indépendance plus faibles et à un manque d'activité physique (Porto et al., [2015](https://www.ncbi.nlm.nih.gov/pubmed/26170652)). Plus récemment, Porto et al. ([2017](https://www.ncbi.nlm.nih.gov/pubmed/28225476)) ont comparé le contrôle de la posture chez les BPCO (n=93) et chez les sujets en bonne santé (n=39) durant des activités dynamiques et statiques. Ils ont montré que les patients présentant une altération plus importante de l’équilibre corporel avaient une plus grande incidence de chutes, et que l’équilibre corporel était plus affecté avec l’âge chez les patients atteints de BPCO que chez les personnes en bonne santé.

Par ailleurs, la capacité à maintenir la stabilité et l’équilibre est essentielle pour l’indépendance fonctionnelle dans les activités de la vie quotidienne, la mobilité, et pour éviter les chutes. Un équilibre détérioré a été associé à un risque accru de chutes entraînant un taux de mortalité plus élevé chez les personnes âgées (AGS/BGS/AAO, [2001](https://www.ncbi.nlm.nih.gov/pubmed/11380764)). Les chutes sont un problème de santé important qui a des conséquences importantes pour les personnes âgées. Il a été estimé que 30 à 50% des personnes de plus de 65 ans tombent au moins une fois par an (Aspray et al., [2006](https://www.ncbi.nlm.nih.gov/pubmed/16364932)). Tinetti et al. ([1988](https://www.ncbi.nlm.nih.gov/pubmed/3205267)) ont défini une chute comme « un événement qui entraîne une personne qui se repose involontairement au sol ou à un niveau inférieur, non pas à la suite d'un événement intrinsèque majeur ou d'un hasard accablant ». Les facteurs de risque de chutes peuvent être divisés en intrinsèques et extrinsèques. Les facteurs intrinsèques sont liés au patient et comprennent: les maladies chroniques, l'âge avancé, les écarts de marche, la faiblesse musculaire, les médicaments multiples et l'état mental altéré. Les facteurs extrinsèques comprennent les activités dangereuses telles que la marche sur des surfaces glissantes, des chaussures inappropriées, des conditions de vie instables ou des risques environnementaux (AGS/BGS/AAO, [2001](https://www.ncbi.nlm.nih.gov/pubmed/11380764)).

Il est raisonnable de supposer que le risque de chute augmente à mesure que le nombre de facteurs de risque s'accumule. Les caractéristiques physiopathologiques de la BPCO suggèrent que les personnes atteintes de cette maladie présentent de nombreux facteurs de risque qui ont également été identifiés chez les personnes âgées, telles que la faiblesse musculaire, les médicaments multiples, la polyneuropathie (Ozge et al., [2001](https://www.ncbi.nlm.nih.gov/pubmed/11402511)). Les chutes ne sont pas seulement associées à la mortalité et à la morbidité, mais sont liées à un fonctionnement global plus faible et à l'admission anticipée dans les établissements de soins de longue durée. Par conséquent, réduire le risque de chute est un objectif important pour la santé publique (Brown, [1999](http://www.tandfonline.com/doi/abs/10.1080/095939899307775)). Tinetti et al. ([1988](https://www.ncbi.nlm.nih.gov/pubmed/3205267)) ont montré chez des personnes âgées, que le risque de chute était de 8% chez ceux qui n'avaient aucun facteur de risque et augmenté jusqu'à 78% pour ceux qui avaient quatre facteurs de risque ou plus. Les chutes qui ne causent pas de blessures commencent fréquemment une spirale descendante de la peur qui mène à l'inactivité et à la diminution de l'agilité, de la force et de l'équilibre et entraîne souvent une perte d'indépendance dans les activités normales d'auto-soins (Tinetti et al., [1994](https://www.ncbi.nlm.nih.gov/pubmed/8169336)).

D’autre part, l'équilibre corporel est un processus fonctionnel complexe qui implique non seulement le système somatosensoriel, mais aussi le système vestibulaire et les récepteurs visuels, avec la participation des structures afférentes et efférentes. L'équilibre peut être affecté par des changements dans la séquence de l'activation musculaire, le recrutement retardé des muscles synergiques, l'activation des muscles antagonistes, l'activation retardée des réponses posturales et même par des changements dans l'amplitude de la réponse musculaire. Ces changements conduisent à une diminution de la capacité de la personne à détecter et à contrôler les oscillations médilo-latérals et antéro-postérieures du corps de manière statique et pendant le mouvement (Souchard, [2005](http://www.em-consulte.com/rmr/article/157120) ; Moxley et al., [1999](https://www.ncbi.nlm.nih.gov/pubmed/10469937?dopt=Abstract)). La preuve actuelle suggère qu'il existe un déficit significatif dans l'équilibre postural chez les personnes souffrants de BPCO et que la base physiopathologique de ces changements est multifactorielle (Smith et al., [2010](https://www.ncbi.nlm.nih.gov/pubmed/20206529)). Les facteurs de risque de chutes chez les sujets atteints de BPCO comprennent une faiblesse des membres inférieurs, des troubles de l'équilibre, un appauvrissement nutritionnel, une malnutrition, des états affectifs, une déficience cognitive et l'utilisation de médicaments (Roig et al., [2009](https://www.ncbi.nlm.nih.gov/pubmed/19419852?dopt=Abstract)). Plusieurs études suggèrent que le contrôle de l'équilibre antéro-postérieur est altéré chez les personnes atteintes de BPCO sévère qui ont besoin d'oxygène supplémentaire (Roig et al., [2009](https://www.ncbi.nlm.nih.gov/pubmed/19419852?dopt=Abstract) ; Butcher et al., [2004](https://www.ncbi.nlm.nih.gov/pubmed/15286536?dopt=Abstract)).

Tout au long des années, un certain nombre d'instruments ont été développés pour mesurer quantitativement l'équilibre dans la population âgée. Ces instruments de dépistage servent à évaluer la capacité à maintenir l'équilibre et, par la suite, à identifier les individus qui présentent un risque important de tomber dans un proche avenir (Mancini et Horak, [2010](https://www.ncbi.nlm.nih.gov/pubmed/20485226)). Le choix des tests d'équilibre dépend du but de l'évaluation. En recherchant dans la littérature, nous avons constaté que les tests les plus courants utilisés sont : l'échelle d'équilibre de Berg (BBS), Timed Up & Go (TUG) et le test de position de jambe unique (SLS). Souvent, l'échelle de confiance des équilibres d'activités (ABC) est considérée comme une partie de l'évaluation du bilan clinique (Alexander, [1994](https://www.ncbi.nlm.nih.gov/pubmed/8277123) ; Berg et Norman, [1996](https://www.ncbi.nlm.nih.gov/pubmed/8890112) ; Bobannon, [2006](http://journals.lww.com/topicsingeriatricrehabilitation/Abstract/2006/01000/Single_Limb_Stance_Times__A_Descriptive.10.aspx); Mancini et Horak, [2010](https://www.ncbi.nlm.nih.gov/pubmed/20485226)). Par ailleurs, le test de marche de 6 minutes (6MWT) fournit une bonne estimation de la capacité aérobie/endurance et de la performance fonctionnelle globale (Bean et al., [2002](https://www.ncbi.nlm.nih.gov/pubmed/12403805)). Les propriétés de mesure de ce test ont bien été établies dans la population du BPCO (Solway et al., [2001](https://www.ncbi.nlm.nih.gov/pubmed?term=%22Chest%22%5BJournal%5D%20AND%202001%5BPDAT%5D%20AND%20119%5BVOL%5D%20AND%20256-70%5BPAGE%5D) ; ERS/ATS, [2014](https://www.ncbi.nlm.nih.gov/pubmed/?term=An+official+European+Respiratory+Society%2F+American+Thoracic+Society+technical+standard%3A+field+walking+tests+in+chronic+respiratory+disease)). Ce test pourrait être associé à l’évaluation de l’équilibre et de la stabilité posturale chez les patients atteints de BPCO. À notre connaissance, les relations entre l’équilibre postural et les marqueurs de la bronchopneumopathie chronique obstructive et notamment les pressions respiratoires maximales, les effets de l’entraînement des muscles inspiratoires, seul et combiné à un programme de réhabilitation respiratoire sur l’équilibre posturale chez les patients souffrants de BPCO, n’ont pas été étudié.

**Approche expérimentale**

Toutes les mesures seront effectuées avant et après le programme d’entraînement. Au niveau respiratoire, nous mesurons la pression inspiratoire maximale (PI_max_) et les mesures spirométriques. Au niveau de l’équilibre, nous procèderons par : test de position de jambe unique, timed Up & Go et les questionnaires ABC et le Berg Balance Scale (Crisan et al., [2015](https://www.ncbi.nlm.nih.gov/pubmed/25768731)). Au niveau de l’effort, nous utiliserons le test de marche de 6 minutes (le CR-10, le RPD, la fréquence cardiaque, la saturation en oxygène et la ventilation seront mesurés lors de ce test).

Nous envisagerons deux groupes de 12 patients minimum. Groupe 1 fera l’entraînement des muscles inspiratoires combiné à l’entraînement en endurance. Groupe 2 sera groupe contrôle, fera seulement l’entraînement en endurance.

Les programmes d’entraînement seront menés sur 8 semaines.

**Sujets**

Les patients seront admis à l'étude s'ils avaient une BPCO diagnostiquée selon les critères de la société américaine Thoracic Society / European Respiratory Society (Vestbo et al., [2013](https://www.ncbi.nlm.nih.gov/pubmed/22878278)). Ils seront divisés en 2 groupes, groupe expérimentale versus un groupe contrôle. Seuls les patients ayant un volume expiratoire forcé dans 1 s (VEMS) <80% de valeur prédite et VEMS / capacité vitale forcée (CVF) <70%) seront admissibles à participer à l'étude. Les critères d'exclusion consistent en (1) troubles psychiatriques ou cognitifs diagnostiqués, (2) troubles neurologiques ou neuromusculaires progressifs, (3) problèmes orthopédiques graves ayant un impact majeur sur les activités quotidiennes et (4) inclusion préalable dans un programme de réadaptation (<1 an) (Charususin et al., [2013](https://www.ncbi.nlm.nih.gov/pubmed/?term=Inspiratory+muscle+training+protocol+for+patients+with+chronic+obstructive+pulmonary+disease+(IMTCO+study)%3A+a+multicentre+randomised+controlled+trial) ; Beaumont et al., [2018](https://www.ncbi.nlm.nih.gov/pubmed/29371379)).

**Entraînement des muscles inspiratoires**

Les patients seront randomisés à l’entraînement des muscles inspiratoires. L’entraînement sera effectué une fois par jour pendant 7 jours durant 8 semaines. Pour l’entraînement des muscles inspiratoires, nous utiliserons un dispositif (PowerBreathe® Medic, IMT Technologies Ltd, Birmingham, UK). L’entraînement consiste à faire 2 séries journalières de 30 inspirations entre coupé de 5 à 10 minutes de récupération (Langer et al., [2015](https://www.ncbi.nlm.nih.gov/pubmed/?term=Efficacy+of+a+Novel+Method+for+Inspiratory+Muscle+Training+in+People+With+Chronic+Obstructive+Pulmonary+Disease)). Avec une charge de 50% de PI_max_ de base, incrémental de 10% de PI_max_ chaque 2 semaines d’entraînement (50%, 60%, 70% et 80% PI max) (Ambrosino, [2018](https://www.ncbi.nlm.nih.gov/pubmed/29371389)). Les patients seront instruits à mettre l’accent sur l’utilisation de leurs diaphragmes et de s’assurer que leurs abdomens « se détachent » au cours de chaque manœuvre inspiratoire (Ramsook et al., [2016](https://www.ncbi.nlm.nih.gov/pubmed/?term=Diaphragm+Recruitment+Increases+during+a+Bout+of+Targeted+Inspiratory+Muscle+Training)). Ils seront familiarisés avec la respiration diaphragmatique en s’asseyant debout sur une chaise et en plaçant une main sur l’abdomen et l’autre main sur les côtes le long de la ligne axillaire antérieure. Les sujets respirent alors et tentent de garder leur main sur la côte stationnaire et ne font que déplacer leur abdomen (Ramsook et al., [2016](https://www.ncbi.nlm.nih.gov/pubmed/?term=Diaphragm+Recruitment+Increases+during+a+Bout+of+Targeted+Inspiratory+Muscle+Training)). Il a été montré que cette méthode induit une augmentation de la participation du diaphragme pendant la respiration naturelle entraînant une amélioration de la capacité fonctionnelle chez les BPCO lors d’un programme d’entraînement respiratoire mettant l’accent sur l’utilisation du diaphragme (Yamaquti et al., [2012](https://www.ncbi.nlm.nih.gov/pubmed/22464088)).

**Entraînement en endurance**

L’entraînement en endurance sera mené pendant 8 semaines en raison de 3 jours par semaine (IMT et groupe contrôle). Le processus de la séance comprendra un exercice aérobie sur un tapis roulant (30 min par jour) puis un stretching des membres supérieurs et inférieurs (Beauchamp et al., [2010](https://www.ncbi.nlm.nih.gov/pubmed/20801268)).

**Mesures**

Les mesures suivantes seront complétées avant et après le programme d’entraînement.

**Pressions respiratoires maximales**

La pression inspiratoire maximale (PI_max_) qu’un sujet peut produire au niveau de la bouche est un indice simple permettant d’évaluer la force des muscles inspiratoires (ATS/ERS, [2002](https://www.ncbi.nlm.nih.gov/pubmed/12186831)). Les mesures seront effectuées à partir du volume résiduel pour la pression inspiratoire maximale (PI_max_), à l’aide d’un appareil portable Manomètre (MicroRPM, MicroMedical Ltd, Kent, Royaume Uni) conformément à la déclaration ATS/ERS ([2002](https://www.ncbi.nlm.nih.gov/pubmed/12186831)). Les évaluations seront répétées au moins cinq fois (récupération de 30 s entre les tentatives), et devraient être poursuivies jusqu'à ce qu'une reproductibilité au moins soit obtenue à partir des trois meilleures mesures (dans une différence de 10 cm H_2_O entre les mesures) (Charususin et al., [2013](https://www.ncbi.nlm.nih.gov/pubmed/?term=Inspiratory+muscle+training+protocol+for+patients+with+chronic+obstructive+pulmonary+disease+(IMTCO+study)%3A+a+multicentre+randomised+controlled+trial)).

**Test spirométrie**

Les manœuvres respiratoires seront effectuées à l’aide d’un spiromètre (EsayOne Diagnostic, Switzerland) (Barr et al., [2008](https://www.ncbi.nlm.nih.gov/pubmed/18364054)). Les variables ventilatoires suivantes seront mesurées au moins trois fois et seules les valeurs reproductibles seront mémorisées. Les tests de l’EFR seront réalisés avant le programme d’entraînement.

- **Capacité vitale forcée (exprimée en litre) : CVF**

Elle correspond au volume de gaz expiré au cours d’une expiration forcée complète et en partant de la position d’inspiration maximale. On peut sous-estimer la CVF si on ne laisse pas au sujet un temps suffisant pour vider ses poumons à bas volume, c'est-à-dire au stade ou le débit expiratoire est déterminé par le mécanisme de limiteur de débit.

- **Volume expiré maximum seconde (exprimé en litre) : VEMS**

Il est obtenu après une inspiration maximale suivie d’un bref temps d’apnée. Le sujet doit effectuer une expiration maximale forcée et rapide. Le VEMS dépend de l’effort musculaire et de la coopération du sujet. C’est un paramètre très largement utilisé et reproductible.

- **Rapport de Tiffeneau = (VEMS/CVF×**
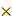
**100)**

Le rapport VEMS/CVF permet d’identifier le pourcentage de la capacité vitale mobilisable en une seconde. Le syndrome ventilatoire obstructif (SVO) est défini par un rapport VEMS/CVF inférieur à 0,7. La sévérité du SVO est déterminée par la valeur du VEMS exprimé en pourcentage de la valeur théorique (Quanjer et al., [1993](https://www.ncbi.nlm.nih.gov/pubmed/?term=Eur+Respir+J+Suppl.+1993+Mar%3B16%3A85-100)). La réversibilité de la fonction respiratoire est vérifiée de la façon suivante : 15 minutes après avoir inspiré quatre bouffées d’un B2- mimétique (Ventoline), le sujet réalise une nouvelle mesure : la réversibilité est reconnue si l’évolution du VEMS après la prise du broncho-dilatateur est supérieure à 12 % de la valeur initiale (Meslier et al., [1989](https://www.ncbi.nlm.nih.gov/pubmed/?term=Eur+Respir+J+1989%3B+2%3A497-505.))**.**

**Berg Balance Scale**

Nous avons choisis le [BBS](https://www.has-sante.fr/portail/upload/docs/application/pdf/Evaluation_%20fonctionnelle_%20AVC_ref.pdf) (Berg et al., [1992](https://www.ncbi.nlm.nih.gov/pubmed/?term=Can+J+Public+Health+1992%3B83(Suppl+2)%3AS7-11.)) comme principal résultat pour cette étude, car il s’agit de la mesure clinique de l’équilibre la plus largement acceptée et psychométriquement robuste pour les personnes âgées (Tyson et Connell, [2009](https://www.ncbi.nlm.nih.gov/pubmed/?term=Clin+Rehabil+2009%3B23%3A824-40.)) et considéré comme le test gold standard pour les capacités d’équilibres statiques et dynamiques (Langley et Mackintosh, [2007](http://nsuworks.nova.edu/ijahsp/vol5/iss4/13/)). Les activités telles que les transferts, l’atteinte, le retournement et l’attitude à une seule jambe ont été classées sur une échelle allant de 0 (incapable / non sécurisé) à 4 (indépendant / efficace / sûr), avec des scores plus élevés indiquant un meilleur contrôle de l'équilibre.

La mesure obtenue à l'aide du BBS a montré une cohérence interne, une fiabilité intra et intergroupes, une validité de contenu, une validité de construction et une validité prédictive pour déterminer le risque de chute chez les personnes âgées (Finchet al., [2002](https://www.ncbi.nlm.nih.gov/nlmcatalog/101175542)). Un score de seuil de 46 et moins a été identifié comme un score utile pour identifier avec succès les personnes à risque de chute (Lajoie et Gallagher, [2004](https://www.ncbi.nlm.nih.gov/pubmed/?term=Arch+Gerontol+Geriatr+2004%3B38%3A11-26.) ; Shumway et al., [1997](https://www.ncbi.nlm.nih.gov/pubmed/9256869)). Un changement de 3,3 (ou ≥ 4 points) a été suggéré de représenter un changement de détection minimal (MDC) chez les patients âgés avec des scores basiques de BBS de 45 à 56 points. Les scores MDC pour les sujets ayant un BBS basique inférieur varient de 5 à 6 points pour les personnes âgées vivant dans un milieu communautaire (Donoghue et al., [2009](https://www.ncbi.nlm.nih.gov/pubmed/?term=J+Rehabil+Med+2009%3B41%3A343-6.)).

**Timed Up & Go**

Nous avons choisis le test TUG pour fournir une mesure chronométrée de l’équilibre et de la mobilité fonctionnelle chez nos patients (Podsiadlo et Richardson, [1991](https://www.ncbi.nlm.nih.gov/pubmed/1991946)). Le test nécessite que le patient se met debout d'un fauteuil standard, marcher 3 m à un rythme confortable, retourner à la chaise et s'asseoir. Un essai sera effectué (non enregistré) où les individus seront autorisés à utiliser une aide à la marche si nécessaire. Le TUG a une grande fiabilité et une validité prédictive intra et intergroupes pour les chutes chez les adultes vivant en milieu communautaire (Finchet al., [2002](https://www.ncbi.nlm.nih.gov/nlmcatalog/101175542) ; Podsiadlo et Richardson, [1991](https://www.ncbi.nlm.nih.gov/pubmed/1991946)). Un score de seuil de 16 secondes ou plus a prédit des chutes chez des personnes âgées vivant dans une résidence communautaire (Okumiya et al., [1998](https://www.ncbi.nlm.nih.gov/pubmed/9670889)). Les scores MDC rapportés varient de 4 secondes chez les patients atteints de la maladie d’Alzheimer (Ries et al., [2009](https://www.ncbi.nlm.nih.gov/pubmed/?term=Phys+Ther+2009%3B89%3A569-79.)) jusqu’à 15 secondes chez les patients âgées fragiles (Nordin et al., [2006](https://www.ncbi.nlm.nih.gov/pubmed/?term=Phys+Ther+2006%3B86%3A646-55.)). Le TUG est un test valide et réactive dans la BPCO (Mesquita et al., [2016](https://www.ncbi.nlm.nih.gov/pubmed/27165963)).

**Test de position de jambe unique**

Le SLS (Single Limb Stance Times) un test d’équilibre statique qui enregistre le temps qu’un participant peut supporter sur une jambe sans assistance. Le SLS sera effectué 3 fois avec une pause entre les répétitions et la meilleure valeur sera utilisée. Le SLS sera établit avec yeux ouverts. Dans une méta-analyse descriptive, Bohannon et al. ([2006](http://journals.lww.com/topicsingeriatricrehabilitation/Abstract/2006/01000/Single_Limb_Stance_Times__A_Descriptive.10.aspx)) ont présenté les normes appropriées pour le SLS.

**L’échelle ABC**

L’échelle [ABC](http://www.archives-pmr.org/article/S0003-9993(06)01318-9/pdf) exige que les patients indiquent leur confiance dans l'exécution de 16 activités sans perdre leur équilibre ou devenir instable sur une échelle de 11 points (0% à 100%) (Myers et al., [1998](https://www.ncbi.nlm.nih.gov/pubmed/?term=J+Gerontol+A+Biol+Sci+Med+Sci+1998%3B53%3A+M287-94.)). Chaque élément décrit une activité spécifique qui nécessite un contrôle de l'équilibre progressivement augmenté. Les résultats les plus élevés indiquent une plus grande confiance dans l'équilibre ou moins de peur de tomber. L'échelle ABC a une bonne fiabilité test-retest, une cohérence interne et une capacité prédictive pour les chutes chez les personnes âgées qui résident dans un milieu communautaire (Finchet al., [2002](https://www.ncbi.nlm.nih.gov/nlmcatalog/101175542) ; Myers et al., [1998](https://www.ncbi.nlm.nih.gov/pubmed/?term=J+Gerontol+A+Biol+Sci+Med+Sci+1998%3B53%3A+M287-94.)). Un changement de 13% a été démontré pour refléter un changement de détection minimal (MDC) pour cette mesure (Steffen et Seney, [2008](https://www.ncbi.nlm.nih.gov/pubmed/?term=Phys+Ther+2008%3B88%3A733-46.)).

**Test de marche 6 minutes**

Le 6MWT est un test valide, réactif, interprétable et autoévalué qui quantifie la capacité d'exercice fonctionnelle en termes de distance parcourue en 6 minutes chez les patients atteints de BPCO (Burge et al., [2000](https://www.ncbi.nlm.nih.gov/pubmed/?term=BMJ+2000%3B320%3A1297-303.)). Le test sera effectué sur un parcours droit de 40 mètres dans un couloir fermé conformément au protocole décrit par la ERS/ATS ([2014](https://www.ncbi.nlm.nih.gov/pubmed/?term=An+official+European+Respiratory+Society%2F+American+Thoracic+Society+technical+standard%3A+field+walking+tests+in+chronic+respiratory+disease)). Au cours de ce test, les paramètres ventilatoires seront évalués par un Spiropalm (COSMED Spiropalm, Rome, Italie) (Ponomareva et al., [2015](http://erj.ersjournals.com/content/46/suppl_59/PA1029)). Au cours de chaque 6MWT, les patients auront des instructions et des encouragements normalisés. Deux tests seront effectués pour tenir compte des améliorations possibles résultant de la familiarisation, avec la plus grande distance enregistrée. Chaque 6MWT sera séparé par un minimum de 30 minutes. Les propriétés de mesure de ce test sont bien établies dans la population du BPCO (Solway et al., [2001](https://www.ncbi.nlm.nih.gov/pubmed?term=%22Chest%22%5BJournal%5D%20AND%202001%5BPDAT%5D%20AND%20119%5BVOL%5D%20AND%20256-70%5BPAGE%5D) ; ERS/ATS, [2014](https://www.ncbi.nlm.nih.gov/pubmed/?term=An+official+European+Respiratory+Society%2F+American+Thoracic+Society+technical+standard%3A+field+walking+tests+in+chronic+respiratory+disease)). Une échelle de la perception du niveau de la dyspnée (RPD : 0-10) (Wilson et Jones, [1991](https://www.ncbi.nlm.nih.gov/pubmed/?term=Long-term+reproducibility+of+Borg+scale+estimates+of+breathlessness+during+exercise.)) sera utilisée après chaque épreuve.

# Analyses statistiques

En supposant un écart-type commun de 4,7 points dans le changement de BBS entre les mesures pré et post-intervention dans le groupe témoin et les groupes d'intervention qui ont été rapportés par Beauchamp et al. [2013](https://pubmed.ncbi.nlm.nih.gov/23975185/), avec un risque d'erreur de type 1 (α) < 5% et le risque d'erreur de type 2 (β) de 80%. Nous avons déterminé une taille d'échantillon de 24 (12 pour chaque groupe) pour détecter une différence entre les groupes dans les évaluations d'équilibre. En considérant un risque d'erreur de type 2 (β) de 90%, nous avons déterminé une taille d’échantillon de 32 (16 pour chaque groupe).

Les statistiques descriptives seront exprimées en moyenne ± ET. Toutes les analyses statistiques seront effectuées à l'aide du logiciel SPSS pour Windows, version 20 (IBM Corp., Armonk, NY, USA). La distribution de normalité sera vérifiée à l'aide du test «Shapiro-Wilk». Lorsque les variables obéissent à la distribution normale, les données seront comparées à l'aide de tests t paramétriques pour les échantillons appariés et les échantillons non appariés, respectivement intra et intergroupes. Sinon, les données seront comparées en utilisant le test non paramétrique «Wilcoxon» pour les échantillons appariés et le test non paramétrique «Mann-Whitney» pour les échantillons non appariés, respectivement intra et intergroupes. La limite de signification est fixée à P <0,05. Un test du khi carré sera utilisé pour comparer les pourcentages des Gold stades. La taille de l’effet (d de Cohen) sera calculée à l’aide des données à la mesure de base et après l’entraînement. Les valeurs du d de Cohen de 0,2, 0,5 et 0,8 seront interprétées comme petites, modérées et grandes, respectivement. Les paramètres ont été analysés en utilisant le coefficient de corrélation de Pearson.
